# The Influence of Hydrazo and Azo Bonds on the Conformation of New 4-Methyl-3,5-dinitro-2-(2-phenylhydrazinyl)pyridine and Its Azo Derivative—Structural Properties, Vibrational Spectra and Quantum Chemical DFT Calculations

**DOI:** 10.3390/ijms262412106

**Published:** 2025-12-16

**Authors:** Jacek Michalski, Edyta Kucharska, Iwona Bryndal, Lucyna Dymińska, Wojciech Sąsiadek, Anna Pyra, Radosław Lisiecki, Maciej Ptak, Jerzy Hanuza

**Affiliations:** 1 Department of Bioorganic Chemistry, Faculty of Production Engineering, Wroclaw University of Economics and Business, 118-120 Komandorska Str., 53-345 Wrocław, Poland; edyta.kucharska@ue.wroc.pl (E.K.);; 2 Department of Organic Chemistry and Drug Technology, Faculty of Pharmacy, Wroclaw Medical University, 211A Borowska Str., 50-556 Wrocław, Poland; 3 Faculty of Chemistry, University of Wrocław, 14 Joliot-Curie Str., 50-383 Wrocław, Poland; 4 Institute of Low Temperature and Structure Research, Polish Academy of Sciences, 2 Okólna Str., 50-422 Wrocław, Polandm.ptak@intibs.pl (M.P.);

**Keywords:** azo and hydrazo groups, pyridine derivatives, crystal structures, spectroscopic analyses, DFT calculations

## Abstract

A review of studies has shown that aromatic azo and hydrazo derivatives are used in a wide spectrum of fields, including food, pharmaceutical, and cosmetic products, as well as in technical and electronic technologies, which has contributed to the development of new such compounds. In this work, the structures of newly obtained 4-methyl-3,5-dinitro-2-(2-phenylhydrazinyl)pyridine (**4MDNPHP**) and its azo derivative, 4-methyl-3,5-dinitro-2-[(*E*)-phenyldiazenyl]pyridine (**4MDNPAP**), were established by spectroscopic (NMR, IR, Raman, and UV-Vis) and emission studies. Single-crystal X-ray diffraction analysis was used to determine the molecular structure of the studied compounds, and the results were compared with DFT calculations (B3LYP/6-311G(2d,2p)). The collected X-ray data revealed that the crystal of the hydrazo compound (**4MDNPHP**) belongs to the triclinic space group *P*$\bar{1}$ (Z = 2), whereas the crystal of the azo compound (**4MDNPAP**) follows the symmetry of the monoclinic space group *P*2_1_/*n* (Z = 4). Both presented derivatives crystallized with one molecule in the asymmetric unit. Specific properties of the hydrazo bridge C_ϕ_-NH-NH-C_θ_ moiety and its azo counterpart C_ϕ_-N=N-C_θ_ were considered in detail.

## 1. Introduction

Aromatic azo and hydrazo derivatives constitute a crucial category of organic compounds, with applications in various fields [1,2]. These include their utilization as dyes and their incorporation into pharmaceutical formulations [3]. Arylhydrazines are composed of a hydrazine group directly bonded to any aromatic ring. This broad definition of this group allows for compounds with highly diverse structures, containing different rings and substituents in different positions. This directly influences a wide range of properties and biological activities (for example, hypotensive [4], anticoagulant [5], and anticancer [6,7]). Hydrazines can also react with the carbonyl groups of enzymes, making them enzyme inhibitors, for example, monoamine oxidase (MAO) [8]. Azo compounds represent a significant class of artificial dyes and pigments, holding a central position within this domain. Due to their applications in the food, pharmaceutical, and cosmetic industries, azo compounds are regarded as highly versatile pigments and are categorized as FD&C dyes [9]. Beyond their use in coloration, they are also deployed in applications such as molecular memory components, nonlinear optical systems, and photoconductors [10,11]. Furthermore, the study of their biological activities has highlighted anti-inflammatory properties [12,13] and anti-cancer potential [14,15]. Azo compounds are also employed in a range of other applications, including textile dyeing [16,17], optical storage systems [18,19,20], and printing technologies [21,22].

Hydrazine compounds have versatile applications in supramolecular chemistry. They are used in three key areas: as molecular switches, metallosupramolecular assemblies, and sensors, thanks to their unique properties, such as ease of synthesis, stability, and, in particular, the possibility of structural and configurational control, e.g., through E/Z isomerization activated by light or chemical stimuli such as pH. They can also be used to create dynamic and stimuli-responsive metallogrids, as well as in a wide range of sensory applications for the detection of metal cations (such as Zn^2+^, Hg^2+^ or Cu^2+^), anions (such as F^−^, CN^−^ or P_2_O_7_^4−^), and neutral molecules (amines, water or cysteine), exploiting their acidic N–H proton and ability to form hydrogen bonds [23].

Studies on hydrazine-connected, stable, luminescent covalent–organic polymers have shown that this material is stable, highly selective, and capable of detecting explosives with record-breaking sensitivity. The connection of an electron-rich, porous structure with precisely arranged hydrogen-bonding sites can be systematically used in developing a new generation of luminescent polymers for advanced chemosensor applications, e.g., compounds with hydrazine groups can be used as a luminescent sensor for nitro-explosives [24].

Despite their extensive applications, the structural and electronic properties of azo and hydrazo derivatives require further investigation, particularly regarding their stability, spectroscopic characteristics, and molecular interactions. The relationship between molecular structure and physicochemical properties remains a fundamental research problem, especially in the context of their potential biological activity and optoelectronic applications. Addressing this issue is crucial for advancing their practical applications and optimizing their performance in diverse industrial and pharmaceutical contexts.

This work extends our existing research concerning phenyl-pyridine derivatives, specifically focusing on azo and hydrazo compounds [25,26]. The structural composition of newly synthesized 4-methyl-3,5-dinitro-2-(2-phenylhydrazinyl)pyridine (**4MDNPHP**), as well as that of its associated azo derivative, 4-methyl-3,5-dinitro-2-[(*E*)-phenyldiazenyl]pyridine (**4MDNPAP**) has been determined. These findings have subsequently been investigated by employing single-crystal X-ray diffraction analysis and spectroscopic methods incorporating NMR, IR, Raman, and UV-Vis. Additionally, the structural and spectroscopic properties were further examined via Density Functional Theory (DFT) calculations, utilizing the B3LYP/6-311G(2d,2p) functional and basis set.

By integrating experimental and computational approaches, this study aims to provide deeper insights into these derivatives, electronic structure, vibrational characteristics, and stability, contributing to a broader understanding of their functional properties and potential applications.

## 2. Results and Discussion

### 2.1. Synthesis and Crystallization

**Scheme 1:**
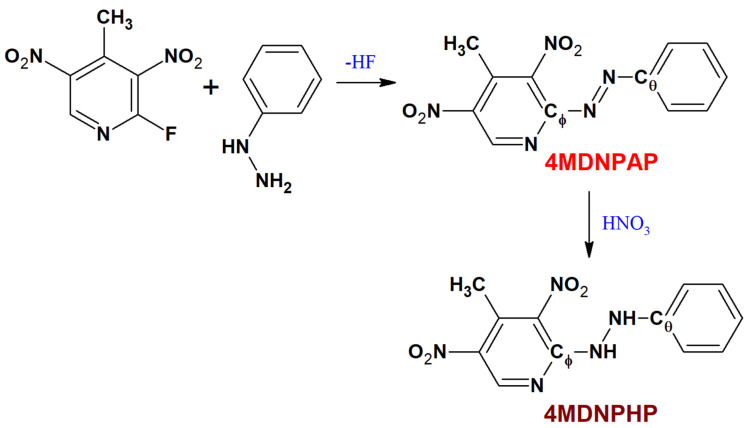
Synthesis of the studied compounds. In the central fragment of the title compounds, carbon atoms belonging to different aromatic rings are marked as: ϕ—pyridine ring and θ—phenyl ring.

The synthesis of the title compounds, **4MDNPHP** and **4MDNPAP**, followed the methodology detailed in a prior publication (refer to Scheme 1) [26]. The yields achieved were 45.3% for **4MDNPHP** and 63.4% for **4MDNPAP**. Furthermore, the resultant materials displayed melting points of 131 °C and 170 °C; correspondingly, **4MDNPHP** presented an orange hue, while **4MDNPAP** manifested as a dark red solid. Purification involved recrystallization; **4MDNPHP** was first treated with ethanol and subsequently with benzene mixed with petrol.

### 2.2. X-Ray Structural Studies

**Figure 1:**
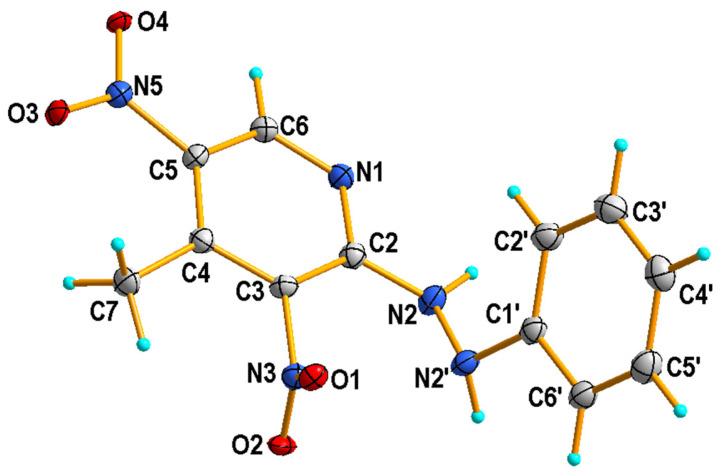
The molecular structure of **4MDNPHP**, showing the atom-numbering scheme and displacement ellipsoids drawn at the 50% probability level.

Selected crystallographic data along with details of structure refinement for **4MDNPHP** and **4MDNPAP** are summarized in Table 1. The hydrazo compound, 4-methyl-3,5-dinitro-2-(2-phenylhydrazinyl)pyridine (**4MDNPHP**), crystallizes in the triclinic space group *P*$\bar{1}$ (*Z* = 2), with one molecule in the asymmetric unit (Figure 1). The geometric parameters obtained from structural analysis (Crystallographic data in Supplementary Materials) and DFT calculations are summarized in Table S1, which is included in the Supplementary Materials of this article. The structure of **4MDNPHP** consists of two rings, pyridine and phenyl, and a central C_ϕ_-NH-NH-C_θ_ moiety linking unit, with the C2-N2-N2’-C1’ torsion angle of −97.41 (9)° (Table S1) and the dihedral angle of 77.9 (2)° between the planes of pyridine and phenyl rings. Such nearly perpendicular conformation, but with an opposite value, has been observed in the case of 2-(2-phenylhydrazinyl)pyridine and its 6-mehyl-3-nitropyridine derivatives studied previously, where the corresponding torsion angle was 85.8 (2)° and 77.3 (2)° [27,28], respectively. Furthermore, the values for this angle [128.1 (2)° and 117.9 (2)°] found in both symmetry-independent molecules of a similar dinitro derivative additionally containing a methyl group at position 6 of the pyridine ring [26] differ significantly from those in **4MDNPHP**.

In contrast to the nitro group attached to the C5 atom, the nitro group attached to the C3 atom is strongly twisted with respect to the pyridine ring plane, resulting in the absence of a typical intramolecular hydrogen bond; the dihedral angles between these planes are 76.1 (2)° and 4.0 (2)°, respectively. So far, a higher value of this angle, involving the nitro group also attached to the C3 atom, has only been observed in 6-methyl-3,5-dinitro-2-[(*E*)-phenyldiazenyl]pyridine [25]. The conformational differences in the **4DNPHP** molecule most likely result from steric hindrance associated with the presence of the -CH_3_ group at position 4 of the pyridine ring, between the two –NO_2_ groups occupying adjacent positions and attached to the C3 and C5 atoms in this ring, next to the hydrazinyl group.

**Figure 2:**
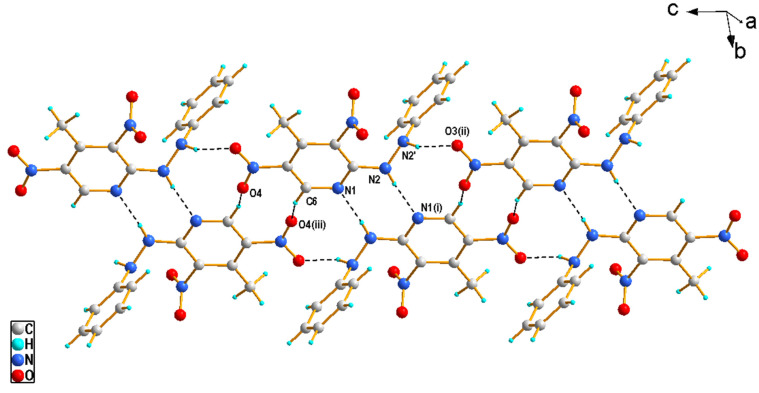
Part of the crystal packing of **4MDNPHP**. The dashed lines (black) indicate intermolecular interactions. Symmetry codes are given in Table 2.

Overall, the crystal structure of **4MDNPHP** exhibits three different types of hydrogen bonds, primarily involving as the -NH-NH- group, the pyridine ring and the nitro group attached to the C5 atom. It should be noted that in **4MDNPHP** there is no intramolecular N2-H2⋯O1 hydrogen bond involving hydrazine (or amino) and nitro groups commonly observed in 2-amino-3-nitropyridine derivatives [26,28,29,30,31]. It seems that the role of the typical intramolecular interaction has most likely been completely taken over by the intermolecular interactions, which are additionally different from those observed in previously studied 3-nitrohydrazinylpyridine derivatives [26,28]. In the **4MDNPHP** crystal, a pair of intermolecular N-H⋯N hydrogen bonds, involving the N2 atom as a donor and the pyridine N1 atom (−*x* + 1, −*y* + 1, −*z*) as an acceptor, connect molecules into a dimer with an R^2^_2_(8) ring motif [32]. These dimers are further linked by the intramolecular N-H⋯O hydrogen bond between the N2’ atom (as a donor) and the nitro O3 atom (*x*, *y*, *z*−1) (as an acceptor), thus forming a double chain propagated in the direction of *c* (Figure 2). The crystal structure of **4MDNPHP** is also stabilized by a pair C-H⋯O interactions formed between the aromatic C6 atom as a donor and the nitro O4 atom (−*x* + 1, −*y* + 1, −z + 1) as an acceptor, which completes the ladder motif in the double chain (Figure 2, Table 2).

The further analysis of non-hydrogen bonding interactions [33] indicates that the nitro group attached to the C3 atom is involved in the N-O⋯π[N3-O2∙∙∙Cg(pyridine) contact, with the shortest O∙∙∙π distance of 2.860 Å, than previously observed [26,28,29]. Aromatic ring π–π stacking interactions have also been recognized, but are weak, the centroid–centroid distance between two phenyl rings is as long as 4.026 Å. The remaining shortest intermolecular distances O⋯N between adjacent nitro groups are 3.069 Å and are equal to the sum of the van der Waals radii, which indicates weak contacts O_NO_2__⋯π(N)_NO_2__ type [34]. Furthermore, Hirshfeld surface (HS) analysis and 2D fingerprint plots generated for **4MDNPAP** indicate the participation of oxygen, nitrogen or carbon atoms in the formation of intermolecular interactions [35]. The areas coloured red on the d_norm_-mapped HS correspond to shorter contacts than the sum of van der Waals radii and confirm the interactions mentioned below. Additional confirmation of the presence of weak interactions is the shape index, where, among other things, triangular red-blue regions correspond to intermolecular interactions of the π…π type (Figures S1 and S2).

**Figure 3:**
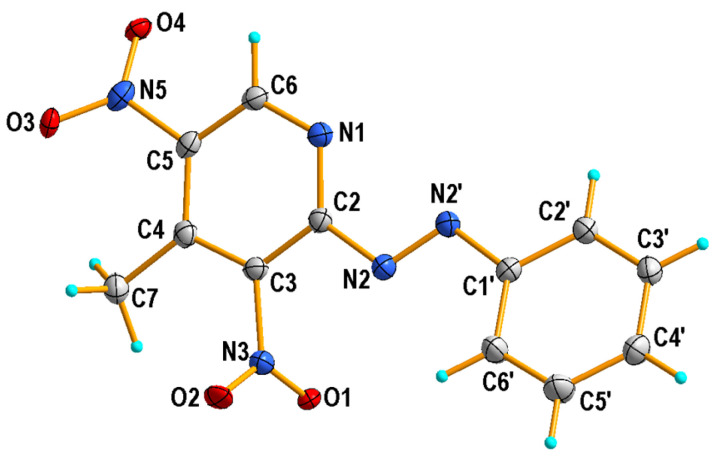
The molecular structure of **4MDNPAP**, showing the atom-numbering scheme and displacement ellipsoids drawn at the 50% probability level.

**Figure 4:**
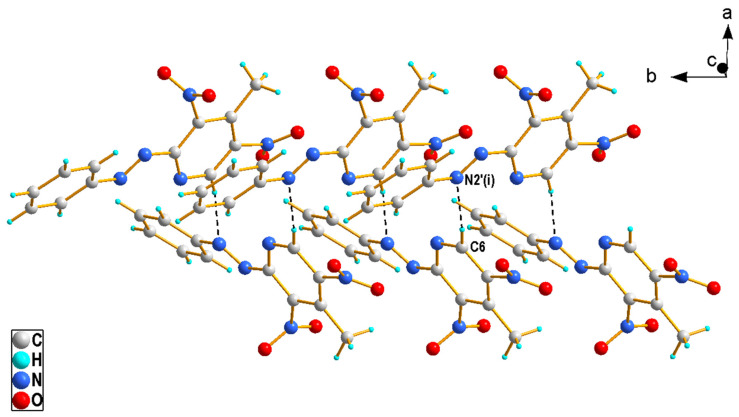
Part of the crystal packing of **4MDNPAP.** The dashed lines (black) indicate C-H⋯N intermolecular interactions. The symmetry code is given in Table 3.

The azo compound, 4-methyl-3,5-dinitro-2-[(*E*)-phenyldiazenyl]pyridine (**4MDNPAP**), crystallizes in the monoclinic space group *P*2_1_/*n* (Z = 4), with one molecule in the asymmetric unit (Figure 3). The structure of **4MDNPHP** consists of two rings, pyridine and phenyl, and a central C_ϕ_-N=N-C_θ_ linking unit that appeared to be in the trans conformation, with the C2-N2-N2’-C1’ torsion angle of 176.49 (12)° (Table S1). It is worth emphasizing, however, that the -N=N- group is not coplanar with the aromatic rings in the **4MDNPAP** molecule, largely due to steric hindrance associated with the presence of four substituents next to each other on the pyridine ring. The dihedral angle of 25.4 (2)° between the planes of the pyridine and phenyl rings is significantly larger than that previously observed in the structures of similar 6-methylpyridine derivatives, where it was much smaller and amounted to 3.5° [25] and 4.8° [36], respectively. As a consequence, the dihedral angles between the mean planes of the C-N=N planes (C_ϕ_-N=N and N=N-C_θ_) and the planes of the phenyl and pyridine rings are 13.0(1)° and 15.0(1)°, respectively, leading to an unexpected conformation of the molecule. Both nitro groups substituted on the pyridine ring are significantly rotated relative to its plane with the dihedral angle between these planes of 74 (2)° and 34.6 (2)° for the NO_2_ group attached to C3 and C5 atoms, respectively. The crystal structure of **4MDNPAP** is stabilized mainly by C-H⋯N interactions (Figure 4, Table 3). The molecules are linked by C-H⋯N interactions involving the aromatic C6 atom as a donor and the hydrazo N2’ atom (−*x* + 3/2, *y* − 1/2, −*z* + 1/2), thus forming a zig-zag chain (Figure 3, Table 3).

The crystal structure of **4MDNPAP** were analyzed in terms of weak C–H⋯O/N,··π⋯π stacking and O_NO_2__⋯π(N)_NO_2__ type, and together with N-O⋯π contacts [33,35]. An analysis revealed that, in addition to the C6-H6⋯N2’ intermolecular interaction, weak C-H⋯O hydrogen bonds exist in the zigzag chain, involving the C7 methyl atom as a donor and the O1 nitro atom (*x*, *y* − 1, *z*) as an acceptor. This interaction is also visible as the red region on the d_norm_-mapped HS, located on the hydrogen methyl (H73) and the oxygen (O1) atoms. Similarly to the **4MDNPHP** crystal, the O⋯N intermolecular distances between adjacent nitro groups are over 3 Å. Furthermore, π–π stacked interactions with d(Cg∙∙∙Cg) < 4.0 Å between the overlapping aromatic rings and short N-O⋯π contacts do not characterize the molecular packing in this case [shortest stacked interactions d(Cg∙∙∙Cg) = 4.9 Å and d(O...π) = 3.65 Å]. An ensemble of HSs and the investigation of 2D fingerprint plots show that the hydrogen and non-hydrogen bonding interactions in the **4MDNPHP** crystal are significantly different from those in the **4MDNPAP** crystal (Figures S3 and S4).

### 2.3. Comparison of Selected X-Ray Crystal Structures from DFT Calculations

The molecular structures of the studied compounds were optimized using initial parameters transferred from their crystal structure. As shown in Table 4 (and also in Table S1, which contains more details for **4MDNPHP** and **4MDNPAP**), the deviations between the calculated and observed geometric parameters are in good agreement. The largest differences concern the values of the torsional angles defining the position of the nitro groups relative to the pyridine ring in both compounds. Moreover, the experimental parameters of the central segment C—N(H)—N(H)—C (C_Φ_-N-N-C_Θ_) show small deviations from the corresponding calculated values in the case of **4MDNPHP**. Since the hydrogen bonds and interactions between molecules inside the crystal structure appeared weak, further modelling of the contacts was not necessary for the calculations.

Comparative analysis of azo and hydrazo analogues indicates that the fundamental geometric differences result primarily from the chemical nature of the bridge—a double N=N bond in azo compounds and a single –NH–NH– bond in hydrazo compounds. In the case of azo analogues, the short N=N bond imposes a planar geometry, which favours effective conjugation with neighbouring aromatic systems and mesomeric stabilization. In the hydrazo analogues, on the other hand, the N–N bond is longer and more flexible, resulting in greater rotational freedom and a wide range of observed torsion angles. In this series, local steric effects (repulsion of nitro and methyl groups) and electrostatic interactions, including potential N–H⋯O(NO_2_) hydrogen bonds and donor–acceptor interactions involving nitro groups, become crucial. This results in different orientations of NO_2_ groups and a lack of clear preference for planar conformers. It can therefore be summarized that the geometry of azo compounds is mainly determined by the requirements of electronic conjugation, whereas in the case of hydrazo, local steric and electrostatic effects play a dominant role, leading to a much greater structural diversity.

### 2.4. Vibrational Spectra

**Figure 5:**
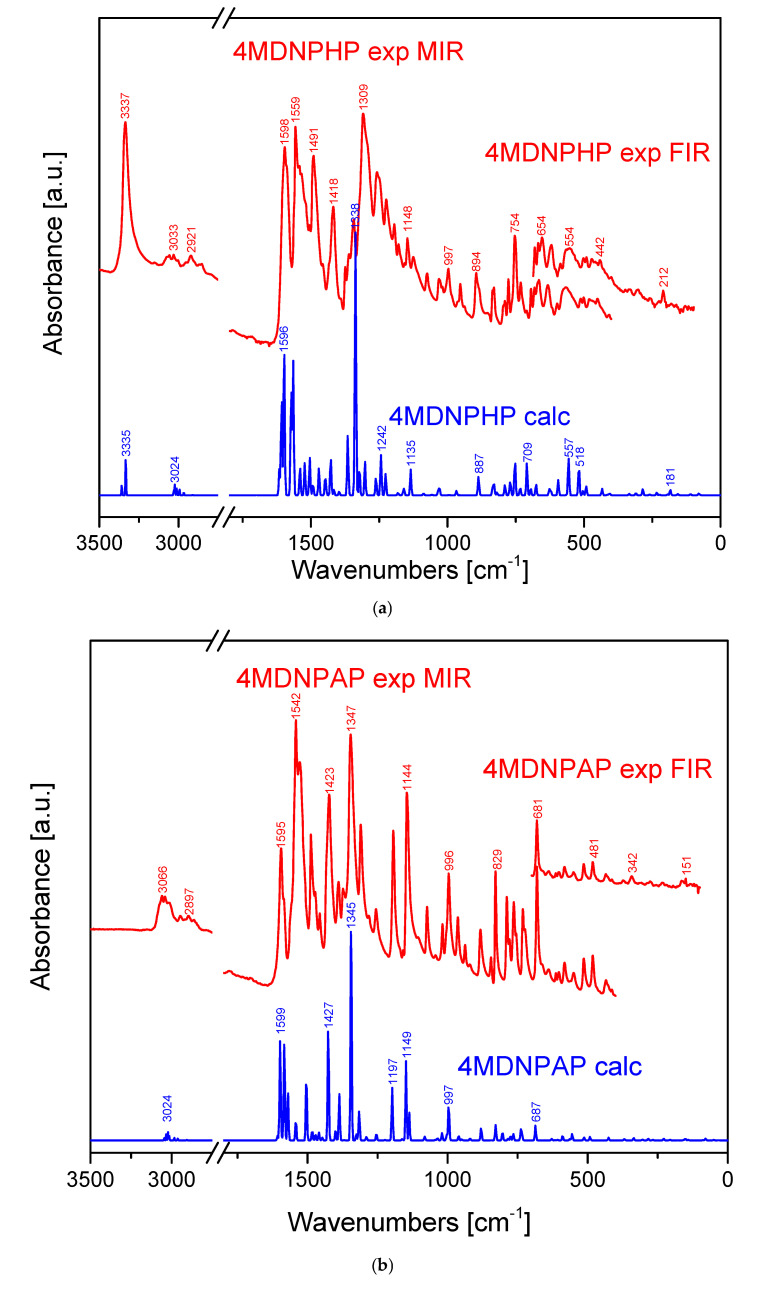

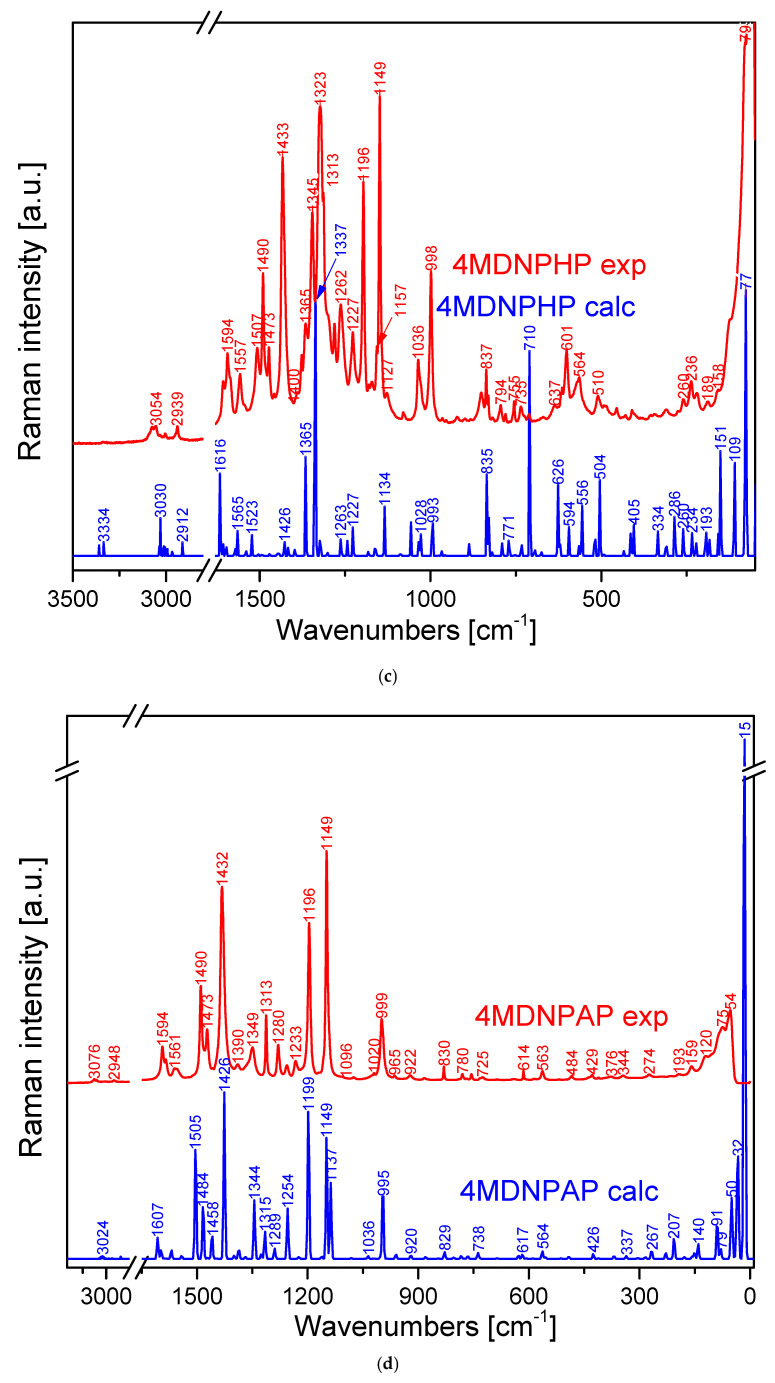
Experimental and calculated IR (**a**,**b**) and Raman (**c**,**d**) spectra of **4MDNPHP** and **4MDNPAP**.

The infrared and Raman spectra of the analyzed compounds (**4MDNPHP** and **4MDNPAP**) are depicted in Figure 5. These spectra reveal characteristic vibrations associated with the pyridine and phenyl rings, alongside methyl and nitro groups, aligning with their established spectral ranges [36,38]. An analysis of the hydrazo and azo compounds and their bonds has been undertaken, specifically in relation to the molecular conformation and the hydrogen bonds identified in the studied crystals. A complete vibrational mode assignment, aligning observed bands to the studied molecules is provided in Table S1. The vibrational properties of both the hydrazo and azo bonds were examined, considering the molecular conformational state and how it interacts with intermolecular hydrogen bonds within the compound. In the studied molecule **4MDNPHP**, the NH group plays a crucial role as a proton donor, participating in intermolecular interactions. The observed vibrational bands in the 3500–2800 cm^−1^ range were assigned to hydrogen bonds of the N–H⋯O type (between the NH and oxygen atoms of the nitro groups) as well as N–H⋯N contacts (involving the NH group and the nitrogen atom of the pyridine ring). The stretching vibration ν(N–H⋯O) located at 3337 cm^−1^ indicates the presence of hydrogen bonds of moderate strength. These results, along with earlier studies, reveal the diversity of hydrogen-bonding interactions among related systems [26,27,31]. For 6-methyl-3-nitro-2-(2-phenylhydrazinyl)pyridine MNPHP [28], the ν(NH) vibrations appear as a doublet at 3318 and 3290 cm^−1^, reflecting the presence of two N–H bonds in the hydrazo bridge. By contrast, the structures reported by Michalski et al. (2013) display a different hydrogen-bonding arrangement [37]. The hydrazo bridge linking bulky substituents in chair-like pyridine conformations enables the formation of two intermolecular N–H⋯N hydrogen bonds between adjacent molecules, creating symmetrical six-membered ring motifs. These interactions give rise to characteristic band splitting in the 3440–3300 cm^−1^ region, attributed to resonance effects between the vibrations of two hydrogen bonds in the ring. In this case, intermolecular hydrogen bonding dominates and significantly enhances crystal stability. For 2-methyl-3,5-dinitro-6-(2-phenylhydrazinyl)pyridine PHPDNM [26], the NH group acts as a proton donor, forming medium-strength intramolecular N–H⋯O hydrogen bonds with nitro oxygen atoms. The characteristic vibrational bands observed in the 3500–2900 cm^−1^ region, with the ν(N–H⋯O) stretching vibration at 3308 cm^−1^, are consistent with the values obtained for **4MDNPHP**, suggesting a similar stabilization mechanism in the solid state.

In summary, **4MDNPHP** exhibits an intermediate character compared to the literature data [26,28,37]. It forms medium-strength intermolecular N–H⋯O interactions and engages in distinct intermolecular N–H⋯N contacts. The coexistence of N–H⋯O and N–H⋯N intermolecular hydrogen bonds stabilizes **4MDNPHP**, highlighting the synergistic role of these interactions in shaping its structural and spectroscopic properties.

**Figure 6:**
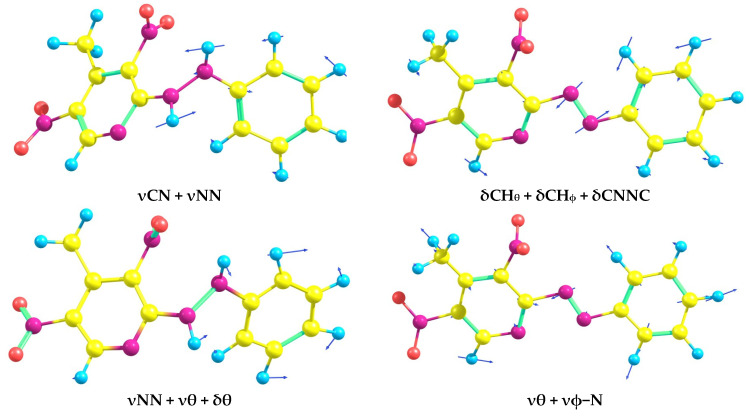
The selected vibrations of the hydrazo and azo bridges, the atomic colors: C—yellow, H—blue, O—red, N—pink.

In **4MDNPHP**, the hydrazo bridge (C_ϕ_–NH–NH–C_θ_, where ϕ = pyridine and θ = phenyl) exhibits a characteristic set of twelve vibrational degrees of freedom, comprising five stretching and seven bending modes. The stretching vibrations are assigned to ν(NH)_ϕ_, ν(NH)θ, ν(C_ϕ_–N), ν(Cθ–N), and ν(N–N), while the bending group includes δ_as_(HN–NH), δ_s_(HN–NH), δ(NNH)_ϕ_, δ(NNH)_θ_, γ(HN–NH), τ(NH–NH), and τ(C_ϕ_N–NC_θ_). The corresponding experimental bands appear at 3337 cm^−1^ [ν(NH)_θ_], 3152 (3301) cm^−1^ [ν(NH)_ϕ_], 1258 (1262) cm^−1^ [ν(C_θ_–N)], 1157 (1146) cm^−1^ [ν(N–N)], and 1031 (1035) cm^−1^ [ν(C_ϕ_–N)], with bending and torsional modes extending from 1589 to 148 cm^−1^: δ_as_(HN-NH) 1589 (1585) cm^−1^, δ(NNH)_ϕ_ 1542 (1544) cm^−1^, δ_s_(HN-NH) 1523 (1533) cm^−1^, δ(NNH)_θ_ 1490 (1490) cm^−1^, γ(HN-NH) 710 (711) cm^−1^, τ(C_ϕ_N-NC_θ_) 230 (237) cm^−1^, and τ(NH-NH) 148 (158) cm^−1^. These data confirm the strong involvement of the hydrazo unit in both stretching and deformation vibrations coupled with motions of the adjacent pyridine and phenyl rings (Figure 6).

A similar vibrational characteristic is observed compared to MNPHP [29]. The bands of the stretching vibrations ν(NH) appear at 3318 and 3290 cm^−1^, and bands of the deformation vibrations in the 1576–1391 cm^−1^ region and torsional/bending modes down to 192 cm^−1^. The data discussed in the earlier paper [37] provide further insight into the variability of hydrazo bond vibrations. The ν(NH) stretching modes are clearly separated into two categories: intermolecular hydrogen-bonded NH stretching vibration (3436–3328 cm^−1^) and intramolecular hydrogen-bonded NH stretching vibration (3265–3168 cm^−1^). The coexistence of intra- and intermolecular hydrogen bonds is corroborated by multiple bands in regions characteristic of bending vibrations, such as δ_as_(HN–NH) and δ_s_(HN–NH). The band of stretching vibration ν(N–N) is observed at slightly higher wavenumbers (1165–1135 cm^−1^) than in **4MDNPHP**, consistent with stronger or more delocalized bonding contributions. In PHPDNM [26], the vibrational characteristic of the hydrazo bridge closely parallels that of **4MDNPHP**. The bands of stretching vibration ν(NH) are observed at 3329 and 3308 cm^−1^, while the ν(N–N) mode at 1144 cm^−1^ and ν(C_ϕ_–N)/ν(C_θ_–N) at 1062 and 1263 cm^−1^ fall in nearly identical positions to those in **4MDNPHP**. The agreement in bending and torsional modes confirms strong coupling of the hydrazo bond with the aromatic fragments, yielding almost identical vibrational characteristics.

Comparison shows that although **4MDNPHP** follows the same 12-mode vibrational scheme as other hydrazo-bonded compounds, the exact band positions and coupling patterns differ. Overall, the spectroscopic characteristics of the hydrazo bond in **4MDNPHP** most closely resemble those of PHPDNM, as described by Michalski et al. (2024) [26].

The bridge, C_ϕ_-N=N-C_θ_, plays an important role in defining the structure and related properties of **4MDNPAP**. The azo ν(N=N) vibration is visible at 1490 cm^−1^ in the Raman and 1487 cm^−1^ in the IR spectrum. The vibrations ν(C_ϕ_=N) and ν(C_θ_=N) of the bridge are assigned to bands at 1194 cm^−1^ (IR) and 1195 cm^−1^ (Raman), respectively. The stretching vibrations of the two C=N bonds, represented as ν_as_(CNNC) and ν_s_(CNNC), create other distinctive vibrations found in the ranges of 1282–1256 cm^−1^ and 1160–1144 cm^−1^, respectively. Further characteristic vibrations for the azo-bond include in-plane bending δ_as_(CNNC) at 550 cm^−1^ and δ_s_(CNNC) at 586–582 cm^−1^, out-of-plane bending γ(CNNC) at 131 cm^−1^, and torsional vibrations τ(CNNC) at 254–246 cm^−1^.

In 6-methyl-3,5-dinitro-2-[(*E*)-phenyldiazenyl]pyridine 6M3,5NPAP [25], the ν(N=N) band is also detected strongly in the Raman spectrum at 1490 cm^−1^. The C–N stretching bands occur at 1199 and 1193 cm^−1^, closely matching 4MDNPAP. The broader vibrational region (1265–1180 cm^−1^) for ν_as_(CNNC) and ν_s_(CNNC) modes demonstrates coupling with pyridine ring vibrations, similar to 4MDNPAP. Characteristic bending and torsional azo modes are observed at slightly different positions: δ_as_(CNNC) 975–910 cm^−1^, δ_s_(CNNC) 640–610 cm^−1^, γ(CNNC) 139–119 cm^−1^, τ(CNNC) 100–30 cm^−1^, suggesting a stronger participation of the azo bridge in low-frequency vibrations compared to **4MDNPAP**.

In 6-methyl-3,5-dinitro-2-[(*E*)-phenyldiazenyl]pyridine (PANP) [26], the band of azo ν(N=N) stretching vibration appears as a weak IR band but a strong Raman band. Two coupled modes are reported: one at 1484/1487 cm^−1^ (IR/Raman) with ~11% of ν(N=N) vibrations, and another at 1469 cm^−1^ with ~44% contribution, confirming resonance effects between azo vibrations and aromatic substituents. The azo bridge is strongly coupled to pyridine, phenyl, and substituents, leading to doublet-like splitting and distribution of N=N character across several normal modes.

All three compounds (**4MDNPAP**, 6M3,5NPAP, PANP) confirm the diagnostic role of the Raman band characteristic of ν(N=N) vibration (1460–1490 cm^−1^), which consistently appears with strong intensity in Raman and weak intensity in IR. For **4MDNPAP**, this band is observed at 1490/1487 cm^−1^, placing it at the higher-frequency edge of the range and suggesting a relatively rigid N=N bond compared to PANP (1469–1487 cm^−1^). The bands characteristic of C–N stretching modes are observed near 1190–1200 cm^−1^ in spectra of three compounds. However, in 6M3,5NPAP [25], they show enhanced sensitivity to substitution effects on the pyridine ring. The greatest variability is found in the low-frequency region: in **4MDNPAP**, the band of τ(CNNC) mode occurs near 254–246 cm^−1^, while in 6M3,5NPAP [25] and PANP [26] the corresponding bands are shifted into the 100–30 cm^−1^ range, reflecting differences in mass distribution and coupling with ring modes. Taken together, these observations demonstrate that **4MDNPAP** shares the same 12-mode vibrational framework of the azo bridge as described for the related compounds 6M3,5NPAP [25] and PANP [26], but also exhibits subtle shifts in band positions and intensities that point to differences in bond strength and delocalization effects associated with substitution [39,40,41,42]. Table 5 compares the characteristic wavenumber values of the nitro group for the azo and hydrazine compounds.

### 2.5. NMR Studies

The ^1^H and ^13^C NMR spectra for both compounds are presented in Figures S5 and S6. Table 6 and Table 7 contain a comparison of the experimental and calculated chemical shifts for **4MDNPHP** and **4MDNPAP**, relative to acetone, with respect to both ^1^H and ^13^C NMR analyses.

Based on the chemical formula unit of the investigated compounds, C_12_H_11_N_6_O_4_ for **4MDNPHP** and C_12_H_9_N_6_O_4_ for **4MDNPAP**, twelve signals would theoretically be expected in the ^13^C NMR spectra. These are associated with six carbon atoms belonging to the benzene ring, five carbon atoms belonging to the pyridine ring and one carbon from a methyl group. The experimental ranges of values, 149.232–114.064 and 153.636–124.895 ppm, correspond to carbons of the benzene ring, respectively, for **4MDNPHP** and **4MDNPAP**. The recorded values for hydrazo and (azo compound), 144.241 (147.346), 154.668 and 154.442, 139.562–135.485 (154.714), and 153.617 (147.751) ppm, correspond to carbons connected with CH_3_, NHNH or NN and two NO_2_ groups, and atom H, respectively, within the pyridine ring structure. The signals at 15.964–14.641 (hydrazo compound) and 24.04 ppm (azo compound) are assigned to the carbon atom of the methyl unit. This specific carbon is situated near three hydrogen atoms and exhibits the largest observed chemical shift. The allocation of the observed spectral signals to specific chemical shifts is consistent with findings in the published literature regarding the NMR spectra of pyridine derivatives [43,44,45,46,47].

The ^1^H NMR spectrum (as seen in Figure S5) of the hydrazo derivative should display eleven lines, while the azo compound is expected to show nine peaks. Concerning the observed chemical shifts, those linked to the hydrogen atoms and appearing at 9.395 and 9.069 ppm in the spectra of the **4MDNPHP** and 9.383 ppm for **4MDNPAP** compounds are associated with the pyridine ring. Resonance signals at 7.993 and 7.749, 7.689, 7.422 and 7.451, and 7.202 ppm for **4MDNPHP** (7.980 and 7.735 ppm for **4MDNPAP**) are indicative of the phenyl ring. The resonances at 2.813, 2.718, and 2.510 ppm for **4MDNPHP** (2.706 ppm for **4MDNPAP**) stem from the methyl groups bound to the pyridine ring at position four. Additionally, the lines at 6.894 and 6.847 ppm are attributable to the protons of the hydrazo group. The chemical shift assignments presented here agree with previous NMR spectral data for similar compounds [48,49,50,51,52].

### 2.6. UV-Vis Studies

**Figure 7:**
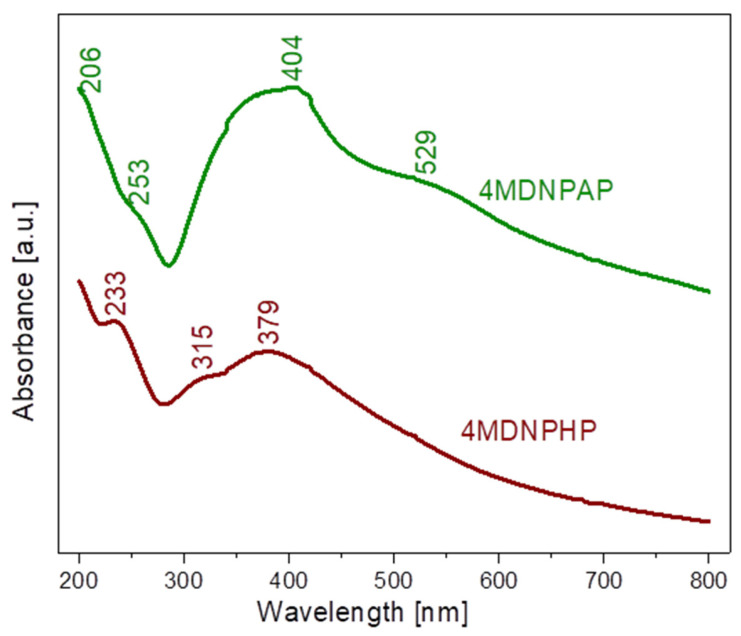
The UV-Vis spectra of **4MDNPHP** and **4MDNPAP**.

**Figure 8:**
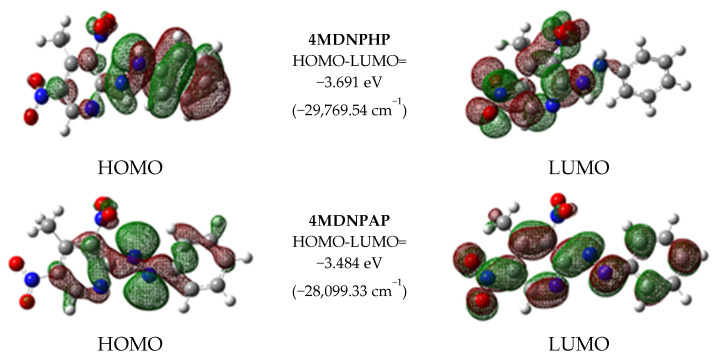
The HOMO-LUMO energy gap of **4MDNPHP** and **4MDNPAP** compounds.

**Figure 9:**
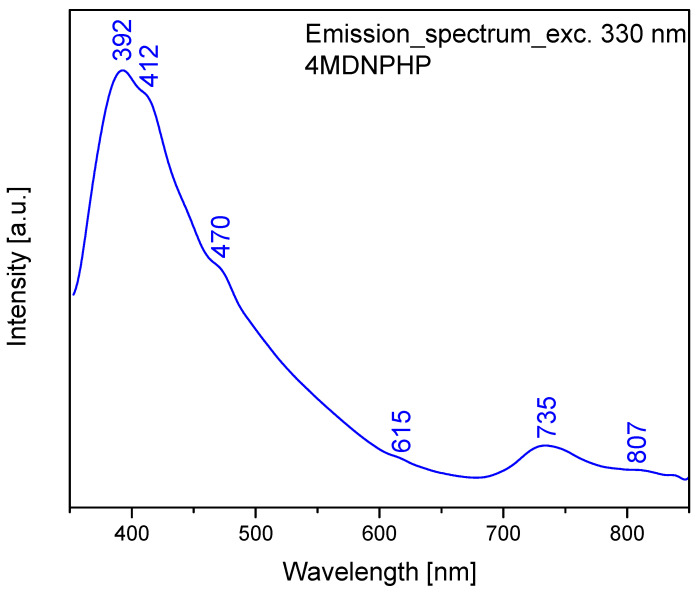
Emission spectrum of **4MDNPHP** excited at 330 nm.

**Figure 10:**
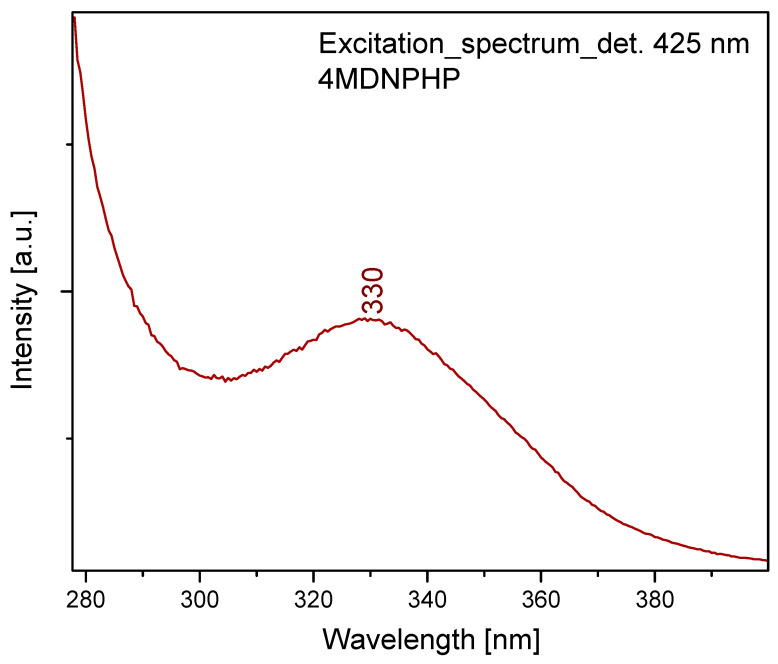
Excitation spectrum of **4MDNPHP** luminescence detected at 425 nm.

**Figure 11:**
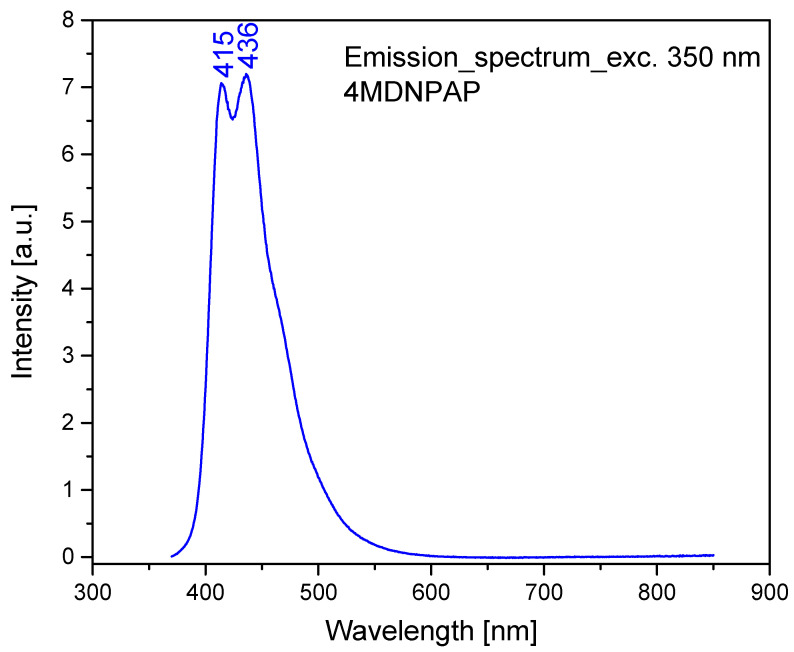
Emission spectrum of **4MDNPAP** excited at 350 nm.

**Figure 12:**
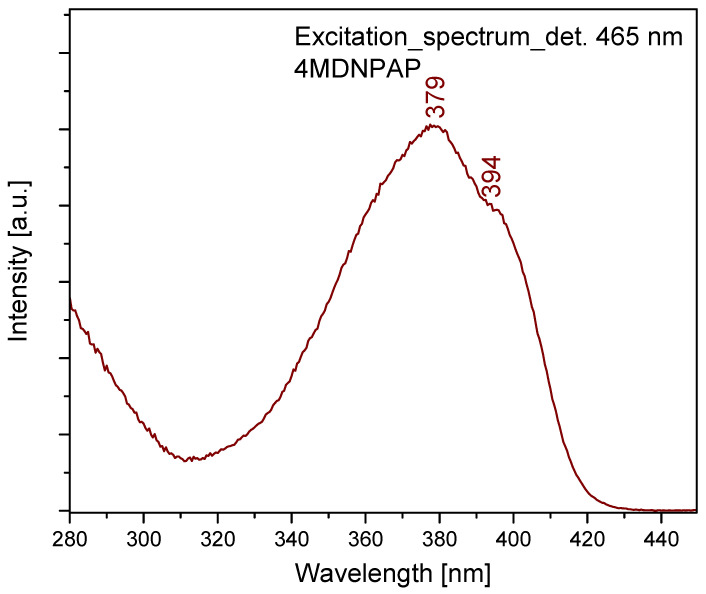
Excitation spectrum of **4MDNPAP** luminescence detected at 465 nm.

The diffuse reflectance spectra are presented in Figure 7 and Figure S7. They reveal a strong absorption band in the range of 230–260 nm (assigned to π-π* transitions between singlet states) and distinct bands in the 360–420 nm range, which are responsible for n-π* transitions. The Gauss deconvolution gives peaks at 203, 314, 372, and 432 nm for **4MDNPHP** and 185, 337, 391, and 495 nm for **4MDNPAP**. They correspond to the S9, S4, S2, and S1 electron levels, respectively. In both spectra, at about 500 nm, a very broad shoulder is observed, which originates from the combination of the S4 and S9 transitions. Calculated singlet and triplet states are collected in Tables S2 and S3, the Mulliken atomic charges estimated for **4MDNPHP** are presented in Tables S4 and S5. The HOMO-LUMO energy gaps shown in Figure 8 correspond to the S0 ⟶  S4 transitions for which the oscillator strengths take values 0.0442 and 0.0550 for **4MDNPHP** and **4MDNPAP**, respectively. The position of the absorption bands agrees with those appearing in the excitation spectra (see Figure 9, Figure 10, Figure 11 and Figure 12), in which the band at 330 nm for **4MDNPHP** and 375–390 nm for **4MDNPAP** are observed. What could have potential application in obtaining these dyes as biosensors [19].

The diffuse reflectance spectra are presented in Figure 7 and Figure S7. They reveal a strong absorption band in the range of 230–260 nm (assigned to π-π* transitions between singlet states) and distinct bands in the 360–420 nm range, which are responsible for n-π* transitions. The Gauss deconvolution gives peaks at 203, 314, 372, and 432 nm for **4MDNPHP** and 185, 337, 391, and 495 nm for **4MDNPAP**. They correspond to the S9, S4, S2, and S1 electron levels, respectively. In both spectra, at about 500 nm, a very broad shoulder is observed, which originates from the combination of the S4 and S9 transitions. Calculated singlet and triplet states are collected in Tables S2 and S3, the Mulliken atomic charges estimated for **4MDNPHP** are presented in Tables S4 and S5. The HOMO-LUMO energy gaps shown in Figure 8 correspond to the S0·⟶··S4 transitions for which the oscillator strengths take values 0.0442 and 0.0550 for **4MDNPHP** and **4MDNPAP**, respectively. The position of the absorption bands agrees with those appearing in the excitation spectra (see Figure 9, Figure 10, Figure 11 and Figure 12), in which the band at 330 nm for **4MDNPHP** and 375–390 nm for **4MDNPAP** are observed. What could have potential application in obtaining these dyes as biosensors [19].

### 2.7. Emission Studies

The emission spectra of the studied compounds are shown in Figure 9, Figure 10, Figure 11 and Figure 12. They showed several key bands, identified by deconvolution into Gaussian components, namely at 390 nm (25,641 cm^−1^), 465 nm (21,505 cm^−1^) and 735 nm (36,054 cm^−1^) for **4MDNPHP** and at 410 nm (24,390 cm^−1^) and 440 nm (22,727 cm^−1^) for **4MDNPAP**. Emission bands are observed in the range 390–735 nm (25,651–36,054 cm^−1^) for **4MDNPHP** and in the range 410–440 nm (24,390–22,727 cm^−1^) for **4MDNPAP**, while electronic transitions in the absorption spectra occur in the ranges 203–432 nm (49,261–23,148 cm^−1^) and 185–495 nm (54,054–20,202 cm^−1^). These results can be explained by a scheme describing the depopulation of excited states. The emission spectra were recorded using excitations at wavelengths of 30,303 (330 nm) and 28,571 cm^−1^ (350 nm), suggesting that the molecules are excited to the S1 state. During the intersystem transition (ISC), the T1, T2, and T3 triplet levels are occupied.

Deconvolution of experimental spectra and DFT calculations were used to elucidate the most probable electronic transitions. The Mulliken charge itself is not a directly observable physical quantity, so it does not directly influence IR or UV-Vis spectra. However, it is an indirectly important theoretical quantity because it reflects the distribution of electron density within the molecule, which in turn influences spectral properties. The influence of Mulliken charges on IR spectra. The IR vibration spectrum depends on: bond strengths (force constants), dipole moments, and their changes during vibration. Because the Mulliken charge shows how electrons are distributed between atoms, greater bond polarization → greater change in dipole moment during vibration leads to greater IR band intensity [53].

These levels were found to be 22,595, 23,555, and 24,092 cm^−1^ for **4MDNPHP** and 12,752, 17,998, and 23,116 cm^−1^ for **4MDNPAP**, respectively (Tables S2 and S3). Strong T1, T2, and T3 ⟶ S0 phosphorescence is observed as broad bands in the 300–700 and 350–600 nm ranges. Although room-temperature phosphorescence is typically not observed in excited triplet states, this explanation for the transition is reasonable given the calculated energies of the molecular orbitals. Their red shift is consistent with expectations. It is well known that intra- and intermolecular hydrogen bonds significantly influence the properties of excited states. Excited-state hydrogen bonds influence non-adiabatic processes such as internal conversion, intersystem transition, intramolecular charge transfer, and photoinduced electron transfer [54,55,56,57,58]. The average Stokes shift for the studied compounds is approximately 3000 cm^−1^.

## 3. Materials and Methods

### 3.1. Materials

All commercially available starting materials (AR grade) obtained from Sigma Aldrich (St. Louis, MO, USA) or Thermo Fischer (Waltham, MA, USA) were used in the synthesis of the studied compounds without prior purification.

### 3.2. X-Ray Structural Studies

Single-crystal X-ray diffraction data for both compounds were collected on an Xcalibur Ruby (Gemini ultra) diffractometer (Rigaku Polska Sp. z o.o., Wroclaw, Poland) at 100 K. The CrysAlisPro software package (Version 1.171.37.35) [59] was used for data collection, cell refinement, data reduction, and analysis. The structures were solved by direct methods using SHELXS-97 [60] and refined by a full-matrix least-squares technique on F^2^ with SHELXL-2013 (and further with SHELXL-2018) [61]. C, N, and O atoms were found in the Fourier difference maps. They were then refined using anisotropic displacement parameters. N-bonded H atoms were freely refined [d(N–H) = 0.87–0.89 (2) Å; U*_iso_*(H) = 1.2 U*_eq_*(N)], while in the final refinement cycles, C-bonded aromatic and methyl H atoms were allowed to rotate and refined in riding mode [d(C–H) = 0.95 Å and 0.98 Å; U*_iso_*(H) = 1.2U*_eq_*(C) and 1.5Ueq(C)]. The figures were prepared using the DIAMOND (Version 4.0) [62] and Mercury (Version 3.8) [63] programs.

### 3.3. IR and Raman Spectra

Room temperature infrared (IR) spectra in the 4000–40 cm^−1^ spectral range, and with the 2 cm^−1^ resolution, were obtained using the KBr pellet method, employing a Biorad 575C FT-IR spectrometer (Bio-Rad Laboratories, Inc., Hercules, CA, USA). Raman spectral data were acquired over the 4000–80 cm^−1^ range, using a Bruker RFS 110/S FT-Raman spectrometer (Bruker Corporation, Billerica, MA, USA) configured in a backscattering geometry and equipped with a YAG:Nd^3+^ laser operating at 1064 nm. The spectra were recorded at a resolution of 2 cm^−1^ and were the product of averaging 64 scans.

### 3.4. NMR Spectra

Proton Nuclear Magnetic Resonance (^1^H NMR) spectra were collected utilizing a JEOL ECZ500R (JEOL Ltd., Tokyo, Japan) instrument operating at 500 MHz. The spectrometer was fitted with a SONDA AUTOMAS 3.2 mm probe, employing a sample rotation rate of 10,000 Hz and the WPMLG (Windowed Phase Modulated Lee-Goldberg) pulse sequence.

### 3.5. UV-Vis Spectra

The diffuse reflectance spectra, ranging from 200 to 1500 nm, were performed at ambient temperature using a Cary-Varian 5E UV-Vis-near-IR spectrophotometer (Agilent Technologies, Santa Clara, CA, USA) equipped with a Praying Mantis diffuse reflectance attachment. The diffuse reflectance spectra were gathered from the powdered samples dispersed in silicon paste; the Al_2_O_3_ powder served as a reference.

### 3.6. Emission Spectra

Emission spectra were collected with an Edinburgh Instruments FLS1000 fluorescence spectrometer (Techcomp Europe Ltd., Livingston, UK). This setup utilized a 450 W xenon lamp for excitation, with a Hamamatsu 928 PMT (Hamamatsu Photonics K.K., Hamamatsu City, Japan) serving as the main detector. The monochromators, configured in the Czerny-Turner mode, incorporated 1800 lines per mm holographic gratings optimized for 300 nm, enabling a 0.2 nm spectral resolution. Spectra obtained were subsequently corrected to account for the sensitivity and wavelength characteristics of the measurement apparatus.

Luminescence decay dynamics were investigated with a femtosecond laser, specifically a Coherent “Libra” model (Coherent Corp., Saxonburg, PA, USA), interfaced with a Light Conversion “OPerA” optical parametric amplifier. Decay curves were acquired utilizing a Princeton Instruments Acton 2500i grating spectrograph (Teledyne Princeton Instruments, Trenton, NJ, USA)coupled to a Hamamatsu C5680 streak camera (Hamamatsu Photonics K.K., Hamamatsu City, Japan). The streak camera offered a temporal resolution of 20 ps and the measurements covered the 200–1100 nm spectral window.

### 3.7. Theoretical Calculations

The molecules of the examined compounds underwent geometry optimization utilizing the Gaussian 16 software suite [64]. The DFT, specifically the B3LYP hybrid functional employing three parameters [65,66,67,68,69], was used in all calculations. The calculations utilized the 6–311G(2d,2p) [70,71] basis set. This choice facilitated comparison with prior findings concerning the optical attributes of other pyridine derivatives, as documented in our previous study [26]. To adjust for vibrational anharmonicity within the calculated wavenumbers, scaling factors of 0.980 (2499–0 cm^−1^) and 0.947 (3500–2500 cm^−1^), determined using the procedure delineated by Palafox and Rastogi [72], were implemented. Following optimization, the geometric parameters, infrared (IR) and Raman wavenumbers, Nuclear Magnetic Resonance (NMR) spectra, and the characteristics of the excited states were calculated for individual molecules. The Potential Energy Distribution (PED) across the normal modes and corresponding internal coordinates was computed using the FCART06 program [73]. The GaussView 6.1 program [74] was employed to visualize the molecular structures. Furthermore, the ChemCraft program (Version 1.8, build 682) [75] served to recalculate the theoretical Raman intensities and visualize specific vibrational modes of the molecules.

## 4. Conclusions

The compound 4-methyl-3,5-dinitro-2-(2-phenylhydrazinyl)pyridine and its azo derivative, 4-methyl-3,5-dinitro-2-[(*E*)-phenyldiazenyl]pyridine, were synthesized and examined using various experimental spectroscopic methods, including ^1^H and ^13^C NMR, IR, Raman, absorption, and emission. The analysis was supported by quantum DFT computations of vibrational, optical, and NMR spectra. Both compounds were further characterized by low-temperature single-crystal X-ray diffraction studies. The **4MDNPHP** compound crystallizes in triclinic symmetry (*P*$\bar{1}$ space group, *Z* = 2), and its hydrazo analogue **4MDNPAP** adopts the monoclinic symmetry (*P*2_1_/*n* space group, *Z* = 4). Their crystal structures, similar to compounds that have shown promising use in medicine, acting as antiviral and antifungal treatments and metal ion complexing agents, were discussed regarding potential applications. In these roles, the molecules bind by using the hydrogen atom of the amino groups or the lone pair electrons of the azo bridge. In the **4MDNPHP** crystal structure, intermolecular interactions, including hydrogen bonding patterns, were analyzed, showing that in **4MDNPHP**, molecules form dimers via N-H⋯N hydrogen bonds, further stabilized by N-H⋯O and C-H⋯O interactions, resulting a ribbon formation. In contrast, **4MDNPAP** exhibits a molecular chain linked through C-H⋯N interactions. In both studied compounds, the adjacent substituents, methyl and -NH-NH-/-N=N- groups, have a significant influence on the geometry of both nitro groups and thus on the entire molecule, its hydrogen bond network or attractive non-covalent contacts. These influence the physicochemical properties and, consequently, their applications in complexing lanthanide ions.

A comparison of azo and hydrazine analogues shows that their geometric differences arise mainly from the nature of the bridging bond. In azo compounds, the short and rigid N=N double bond usually enforces a planar structure, promoting conjugation with aromatic rings. However, that the -N=N- group is not coplanar with the aromatic rings in the **4MDNPAP** molecule, largely due to steric hindrance associated with the presence of four substituents next to each other on the pyridine ring [with the dihedral angle of 25.4 (2)° between the aromatic rings]. On the other hand, in hydrazine derivatives, the longer and more flexible N–N single bond allows free rotation, resulting in a wide range of torsional angles and varied orientations of nitro groups. Their conformations are strongly influenced by steric and electrostatic effects, including possible N–H⋯O(NO_2_) hydrogen bonding, but in the case of **4MDNPHP** the role of typical intramolecular interactions has most likely been completely taken over by intermolecular interactions. Furthermore, hydrazines exhibit greater structural diversity compared to the more rigid and typically conjugated azo systems. These differences translate directly into distinct coordination behaviours toward lanthanide ions. The azo system is predisposed to form more rigid and predictable coordination environments, whereas the hydrazine derivative offers enhanced adaptability due to its conformational freedom and the presence of an additional NH donor site. Consequently, the present study provides clear guidelines for the rational design of azo- and hydrazine-based ligands with tailored structural and functional properties for lanthanide complexation.

Vibrational properties of hydrazine and azo bonds were investigated with respect to molecular conformation and the influence of intermolecular hydrogen bonding. In the **4MDNPHP** molecule, the NH group plays an important role as a proton donor. For the **4MDNPAP** compound, the characteristic ν(N=N) Raman band in the 1460–1490 cm^−1^ range was confirmed, showing high intensity in Raman spectra and low intensity in IR. Strong coupling of the azo bridge with aromatic rings leads to splitting and distribution of the N=N character across several normal vibration modes.

Hirshfeld surface analysis combined with the associated two-dimensional fingerprint plots revealed that both hydrogen-bonding and non-hydrogen-bonding interactions in the **4MDNPHP** crystal differ significantly from those observed in the **4MDNPAP** crystal, further highlighting the substantial influence of the bridging unit on the supramolecular organization.

## Figures and Tables

**Table 1:** Crystallographic data and structure refinement details for the studied compounds.

	4MDNPHP	4MDNPAP
Chemical formula	C_12_H_11_N_5_O_4_	C_12_H_9_N_5_O_4_
*M_r_* (g/mol)	289.26	287.24
Crystal system, space group	triclinic, *P*$\bar{1}$	monoclinic, *P*2_1_/*n*
Temperature (K)	100	100
*a* (Å)	8.006 (2)	8.085 (3)
*b* (Å)	8.735 (3)	6.035 (2)
*c* (Å)	9.518 (3)	26.238 (9)
*α* (°)	104.13 (3)	90
*β* (°)	99.20 (3)	96.26 (3)
*γ* (°)	95.11 (3)	90
V (Å^3^)	631.4 (3)	1272.4 (7)
*Z*	2	4
*λ* (Å)	0.71073	0.71073
*µ* (mm^−1^)	0.12	0.12
Crystal size (mm)	0.50 × 0.36 × 0.26	0.41 × 0.19 × 0.10
No of measured, independent and observed [*I* > 2σ (*I*)] reflections	14,468, 4051, 3714	12,114, 4067, 3242
*R*_int_	0.017	0.044
*θ_max_*, *θ_min_* (°)	31.8, 2.8	32.0, 2.7
*R*_1_ [*F*^2^ > 2σ (*F*^2^)]	0.034	0.055
*wR* (*F*^2^)	0.099	0.135
Goof on F^2^	1.07	1.08
Δ*ρ_max_*, Δ*ρ_min_* (e Å^−3^)	0.45, −0.28	0.51, −0.24
CCDC No	2427161	2427162

**Table 2:** Hydrogen-bond geometry (Å, °) for **4MDNPHP**.

*D*—H⋯*A*	*D*—H	H⋯*A*	*D*⋯*A*	*D*—H⋯*A*
N2—H2⋯N1 ^i^	0.885 (14)	2.121 (14)	3.0042 (14)	174.8 (12)
N2’—H2’⋯O3 ^ii^	0.869 (13)	2.295 (13)	3.1425 (14)	164.7 (11)
C6—H6⋯O4 ^iii^	0.95	2.45	3.2525 (16)	142

Symmetry codes: (i) −*x* + 1, −*y* + 1, −*z*; (ii) *x*, *y*, *z* − 1; (iii) −*x* + 1, −*y* + 1, −*z* + 1.

**Table 3:** Hydrogen-bond geometry (Å, °) for **4MDNPAP**.

*D*—H⋯*A*	*D*—H	H⋯*A*	*D*⋯*A*	*D*—H⋯*A*
C6—H6⋯N2’ ^i^	0.95	2.52	3.460 (2)	173
C7—H73⋯O1 ^ii^	0.98	2.53	3.398 (2)	147

Symmetry codes: (i) −*x* + 3/2, *y* − 1/2, −*z* + 1/2; (ii) *x*, *y* − 1, *z*.

**Table 4:** Comparison of selected experimental and calculated geometric parameters of the studies compounds (**1**, **4**) and their azo analogues (compounds **2**, **3**) and hydrazo analogues (compounds **5**–**10**).

No Compound	-N=N-/-HN-NH- Exp/*calc* [Å, °]				-NO_2_ (p3) Exp/*calc* [°]		-NO_2_ (p5) Exp/*calc* [°]	
	N-N	N-N-C_Φ_	N-N-C_Θ_	C_Φ_-N-N-C_Θ_	C-N-O	C-C-N-O	C-N-O	C-C-N-O
1	1.2562 *1.2524*	114.29 *114.35*	113.68 *115.11*	176.49 *178.44*	117.36 *116.92*	106.19 *97.69*	118.22 *117.94*	34.07 *25.21*
2	1.237 *1.251*	113.2 *113.2*	114.5 *115.6*	−178.6 *−178.6*	116.9 *116.6*	−89.7 *−42.2*	117.2 *117.3*	19.4 *13.7*
3	1.240 *1.279*	114.5 *113.9*	116.1 *115.2*	4.8 *−179.0*	–	–	118.4 *117.4*	– *14.9*
4	1.3953 *1.3852*	119.07 *122.19*	116.60 *117.94*	−97.41 *−112.61*	118.03 *117.86*	−77.90 *−63.92*	119.58 *118.29*	1.79 *22.32*
5	1.391 *1.384*	121.06 *121.59*	116.01 *117.49*	128.10 *150.83*	118.90 *117.99*	170.90 *179.82*	117.77 *117.48*	22.2 *0.70*
6	1.407 *1.381*	119.96 *121.7*	115.02 *118.5*	77.3 −*127.1*	119.20 *118.70*	166.8 *178.6*	–	–
7	– *1.398*	– *120.73*	– *116.79*	– *103.16*	–	–	– *117.53*	– *−0.10*
8	– *1.383*	– *120.68*	– *118.38*	– *144.75*	–	–	– *117.29*	– *−22.48*
9	– *1.399*	*–* *120.43*	*–* *116.78*	*–* *104.20*	–	–	*–* *118.41*	*–* *−1.79*
10	– *1.397*	– *120.85*	– *116.77*	– *1–2.18*	–	–	– *117.58*	– *−0.39*

Explanations: Both azo and hydrazo compounds are composed of a pyridine ring substituted with one or two nitro groups and a methyl group (one is missing). The pyridine ring is connected via an azo or hydrazide bridge from the 2-position to an unsubstituted phenyl ring. Substitution positions in individual **azo** compounds: 2—6-methyl-3,5-dinitro [25], 3—6-methyl-5-nitro [36]. **Hydrazo** analogues: 5—6-methyl-3,5-dinitro [26], 6—6-methyl-3-nitro [28], 7—none methyl-5-nitro [37], 8—3-methyl-5-nitro [37], 9—4-methyl-5-nitro [37], 10—6-methyl-5-nitro [37]; C_Φ_—carbon atom of the pyridine ring, C_Θ_—carbon atom of the phenyl ring; (p3)—position 3, (p5)—position 5.

**Table 5:** The wavenumbers (cm^−1^) corresponding to the vibrations of the NO_2_ groups.

4MDNPAP		4MDNPHP		Mode
IR	Raman	IR	Raman	
1589–1544	1585–1542	1584–1542	1584–1562	ν_as_(NO_2_)
1377	1377–1323	1378–1347	1349	ν_s_(NO_2_)
884–883	882–836	829	830	δ(NO_2_)
777–732	781–735	754	756–726	ω(NO_2_)
107	-	-	-	τ(NO_2_)

**Table 6:** ^1^H NMR experimental and calculated chemical shifts for **4MDNPHP** and **4MDNPAP**.

4MDNPHP			4MDNPAP		
	Chemical Shifts			Chemical Shifts	
No Atom	Exp.	Calc.	No Atom	Exp.	Calc.
H6_ϕ_	9.395, 9.069	9.4247	H6_ϕ_	9.383	9.7110
H3’_θ_	8.915	7.6051	H2’_θ_		8.7666
H5’_θ_	7.993, 7.749	7.5889	H6’_θ_	7.980	8.1696
H7’_θ_	7.689	7.2627	H3’_θ_		8.1100
H6’_θ_	7.442, 7.351	7.0352	H5’_θ_	7.735	7.8670
H2’_θ_	7.202	6.9245	H4’_θ_	7.667	7.8670
H_N_2 (NH)_ϕ_	6.894	6.4176			
H_N_2’ (NH)_θ_	6.847	5.1554			
H7(3) (CH_3_)	2.813	2.7820	H7(3) (CH_3_)		3.0483
H7(2) (CH_3_)	2.718	2.7156	H7(2) (CH_3_)	2.706	2.8735
H7(1) (CH_3_)	2.510	2.1235	H7(1) (CH_3_)		2.3483

**Table 7:** ^13^C NMR experimental and calculated chemical shifts for **4MDNPHP** and **4MDNPAP**.

4MDNPHP			4MDNPAP		
	Chemical Shifts			Chemical Shifts	
No Atom	Exp.	Calc.	No Atom	Exp.	Calc.
C2_ϕ_	154.688, 154.442	163.240	C5_ϕ_	154.714	163.037
C6_ϕ_	153.617	155.303	C2_ϕ_		160.130
C1’_θ_	149.232, 148.678, 147.737	154.319	C1’_θ_	153.636	159.406
C4_ϕ_	144.241	150.910	C3_ϕ_		157.549
C5_ϕ_	139.562, 139.338, 139.236, 138.732	146.711	C6_ϕ_	147.751	154.258
C3_ϕ_	135.485	140.587	C4_ϕ_	147.346	148.557
C3’_θ_	133.761, 133.088	135.400	C2’_θ_	145.610	145.327
C5’_θ_	130.782, 129.826, 129.797, 129.173	135.290	C4’_θ_	139.233	143.995
C4’_θ_	124.887, 123.256	125.668	C3’_θ_	135.489	135.864
C6’_θ_	121.408, 119.525	117.989	C5’_θ_	130.796	135.071
C2’_θ_	114.116, 114.064	115.006	C6’_θ_	124.895	118.367
C7 (CH_3_)	15.964, 15.287, 14.641	20.1131	C7 (CH_3_)	14.651	19.000

(ϕ—pyridine ring, θ—phenyl ring).

## Data Availability

The original contributions presented in this study are included in the article/Supplementary Materials. Further inquiries can be directed to the corresponding author(s).

## Supplementary Materials

The following supporting information can be downloaded at: https://www.mdpi.com/article/10.3390/ijms262412106/s1.
