# Supplementary material for: The Influence of Hydrazo and Azo Bonds on the Conformation of New 4-Methyl-3,5-dinitro-2-(2-phenylhydrazinyl)pyridine and Its Azo Derivative—Structural Properties, Vibrational Spectra and Quantum Chemical DFT Calculations
Source: Int J Mol Sci. 2025 Dec 16;26(24):12106. doi: 10.3390/ijms262412106 (PMC12733332; doi:10.3390/ijms262412106)
Supplement: Supplementary file 1 [file ijms-26-12106-s001.zip › ijms-4004119-supplementary.pdf]

Supporting information

The influence of hydrazo and azo bonds on the conformation of new 4-methyl-3,5-dinitro-2-(2-phenylhydrazynyl)pyridine and its azo derivative – structural properties, vibrational spectra and quantum chemical DFT calculations

Jacek Michalski[1,*], Edyta Kucharska[1], Iwona Bryndal[2], Lucyna Dymińska[1], Wojciech Sąsiadek[1], Anna Pyra[3], Radosław Lisiecki[4], Maciej Ptak[4] and Jerzy Hanuza[4]

Tables S1–S5, Figures S1–S7

Crystallographic data:

4MDNPHP.cif
4MDNPHP_checkcif.pdf
4MDNPAP.cif
4MDNPAP_checkcif.pdf

Table S1. Comparison of selected geometrical parameters (Å, °) of the studied compounds.

	4MDNPHP		4MDNPAP			4MDNPHP		4MDNPAP	
	Exp.	*Calc.*	*Exp.*	*Calc.*		*Exp.*	*Calc.*	*Exp.*	*Calc.*
Bond distances									
N1—C2	1.3485 (12)	1.3392	1.3368 (17)	1.3317	C5'—C6'	1.3862 (13)	1.3856	1.382 (2)	1.3826
N1—C6	1.3269 (11)	1.3200	1.3317 (18)	1.3226	C3—C4	1.3849 (11)	1.3912	1.3886 (19)	1.3905
C2—C3	1.4136 (11)	1.4098	1.387 (2)	1.3965	C3—N3	1.4733 (12)	1.4749	1.4736 (17)	1.4770
C2—N2	1.3511 (10)	1.3649	1.4220 (18)	1.4134	N3—O1	1.2213 (10)	1.2194	1.2174 (17)	1.2193
N2—N2'	1.3953 (12)	1.3852	1.2562 (17)	1.2524	N3—O2	1.2250 (10)	1.2254	1.2176 (16)	1.2194
N2'—C1'	1.4126 (12)	1.4107	1.4189 (18)	1.4100	C4—C5	1.4121 (12)	1.4067	1.3981 (19)	1.4010
C1'—C2'	1.3958 (12)	1.3956	1.390 (2)	1.3974	C5—C6	1.3903 (11)	1.3918	1.388 (2)	1.3947
C1'—C6'	1.3990 (13)	1.3986	1.398 (2)	1.4025	C5—N5	1.4479 (11)	1.4622	1.4610 (18)	1.4703

C2'—C3'	1.3929 (13)	1.3911	1.386 (2)	1.3875	N5—O3	1.2314 (10)	1.2270	1.2298 (17)	1.2251
C3'—C4'	1.3881 (14)	1.3884	1.388 (2)	1.3907	N5—O4	1.2340 (11)	1.2262	1.2258 (17)	1.2233
C4'—C5'	1.3916 (14)	1.3926	1.393 (2)	1.3971	C4—C7	1.5015 (12)	1.5047	1.495 (2)	1.5030
Bond angles									
C2—N1—C6	118.20 (7)	118.24	116.73 (12)	117.53	C2—C3—N3	119.34 (7)	119.49	117.83 (12)	118.03
N1—C2—C3	120.42 (7)	121.24	122.23 (12)	121.71	C4—C3—N3	117.47 (7)	118.70	118.76 (12)	119.14
N1—C2—N2	114.93 (7)	114.46	122.24 (12)	122.33	O1—N3—O2	125.26 (8)	125.55	125.65 (12)	126.43
C3—C2—N2	124.62 (8)	124.29	115.37 (12)	115.90	O1—N3—C3	118.03 (7)	117.86	117.36 (11)	116.92
N2'—N2—C2	119.07 (7)	122.19	114.29 (11)	114.35	O2—N3—C3	116.64 (7)	116.54	116.98 (12)	116.92
N2—N2'—C1'	116.60 (7)	117.94	113.68 (11)	115.11	C3—C4—C5	113.85 (7)	114.64	112.12 (12)	113.29
C6'—C1'—C2'	119.86 (8)	119.50	120.81 (13)	120.30	C4—C5—C6	121.04 (7)	120.57	122.79 (12)	121.33
C6'—C1'—N2'	117.81 (8)	118.47	123.92 (13)	124.71	C4—C5—N5	122.70 (7)	122.82	120.58 (13)	122.04
C2'—C1'—N2'	122.23 (8)	122.00	115.27 (12)	114.99	C6—C5—N5	116.26 (7)	116.61	116.62 (12)	116.62
C3'—C2'—C1'	119.45 (8)	119.74	119.86 (13)	120.00	O3—N5—O4	122.06 (7)	124.32	124.38 (13)	124.89
C2'—C3'—C4'	120.79 (9)	120.87	119.62 (14)	119.69	O3—N5—C5	119.58 (7)	118.29	118.22 (12)	117.94
C3'—C4'—C5'	119.50 (9)	119.19	120.31 (14)	120.37	O4—N5—C5	118.34 (7)	117.38	117.39 (12)	117.16
C4'—C5'—C6'	120.44 (8)	120.57	120.57 (14)	120.34	N1—C6—C5	123.35 (8)	123.41	122.66 (12)	123.29
C5'—C6'—C1'	119.96 (8)	120.12	118.80 (14)	119.31	C3—C4—C7	121.04 (8)	121.46	121.94 (12)	122.56
C2—C3—C4	122.98 (8)	121.72	123.40 (12)	122.83	C5—C4—C7	125.02 (7)	123.81	125.93 (13)	125.47
Torsion angles									
C6—N1—C2—C3	1.34 (11)	2.30	−1.6 (2)	-0.98	C4—C3—N3—O1	−77.90 (10)	-63.92	106.19 (15)	97.69
C6—N1—C2—N2	179.68 (7)	-179.19	−176.84 (12)	-178.25	C2—C3—N3—O1	107.25 (9)	120.00	−74.75 (17)	-82.14
N1—C2—N2—N2'	158.94 (7)	157.53	−15.04 (19)	-18.44	C4—C3—N3—O2	99.47 (9)	113.64	−73.13 (18)	-81.10
C3—C2—N2—N2'	−22.79 (12)	-24.02	169.41 (12)	164.14	C2—C3—N3—O2	−75.38 (10)	-62.44	105.93 (16)	99.07
C2—N2—N2'—C1'	−97.41 (9)	-112.61	176.49 (12)	178.44	C2—C3—C4—C5	−3.49 (11)	1.94	1.89 (19)	-0.02
N2—N2'—C1'—C6'	−164.83 (7)	-156.29	−11.5 (2)	0.37	N3—C3—C4—C5	−178.14 (6)	-174.05	−179.10 (12)	-179.84
N2—N2'—C1'—C2'	18.77 (11)	25.79	169.28 (13)	-179.68	C3—C4—C5—C6	4.51 (11)	2.00	−1.06 (19)	0.16
C6'—C1'—C2'—C3'	−0.65 (12)	-0.15	1.9 (2)	-0.17	C3—C4—C5—N5	−174.76 (7)	-178.07	179.69 (12)	-178.63
N2'—C1'—C2'—C3'	175.67 (8)	177.75	−178.90 (13)	179.87	C4—C5—N5—O3	1.79 (12)	22.32	34.07 (19)	25.21
C1'—C2'—C3'—C4'	0.17 (13)	-0.08	−1.0 (2)	0.12	C6—C5—N5—O3	−177.52 (7)	-157.75	−145.23 (14)	-153.63

C2'—C3'—C4'—C5'	0.30 (13)	0.04	−0.3 (3)	-0.01	C4—C5—N5—O4	−179.94 (7)	-159.08	−146.53 (14)	-155.89
C3'—C4'—C5'—C6'	−0.29 (13)	0.23	0.9 (3)	-0.06	C6—C5—N5—O4	0.75 (11)	20.85	34.17 (18)	25.27
C4'—C5'—C6'—C1'	−0.20 (13)	-0.47	−0.1 (3)	0.01	C2—N1—C6—C5	−0.23 (12)	1.812	2.4 (2)	1.138
C2'—C1'—C6'—C5'	0.67 (12)	0.43	−1.3 (2)	0.11	C2—C3—C4—C7	173.19 (7)	178.47	−179.52 (13)	179.02
N2'—C1'—C6'—C5'	−175.82 (7)	-177.55	179.55 (15)	-179.94	C4—C5—C6—N1	0.67 (12)	-4.095	−1.1 (2)	-0.75
N1—C2—C3—C4	−2.89 (12)	-4.28	−0.6 (2)	0.45	N5—C5—C6—N1	176.42 (7)	175.97	178.17 (12)	178.10
N2—C2—C3—C4	−177.51 (7)	177.36	174.91 (13)	177.88	C6—C5—C4—C7	−172.01 (7)	-174.44	−179.58 (14)	-178.84
N1—C2—C3—N3	175.23 (7)	171.68	−179.66 (12)	-179.73	N3—C3—C4—C7	−1.46 (11)	2.48	−0.5 (2)	-0.80
N2—C2—C3—N3	−2.95 (12)	-6.68	−4.11 (18)	-2.30	N5—C5—C4—C7	8.71 (12)	5.49	1.2 (2)	2.38

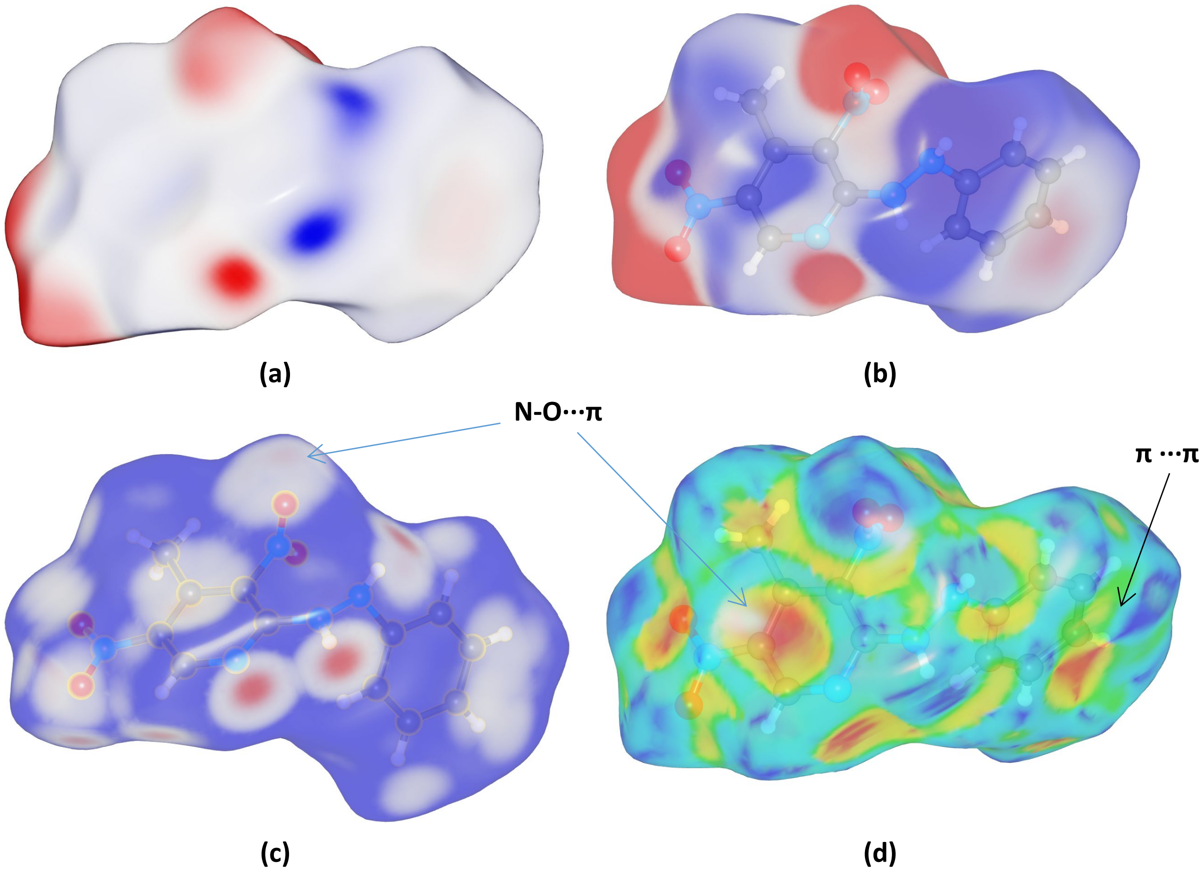

Fig. S1. Hirshfeld surfaces for **4MDNPHP** with: (a) the electrostatic potential (EP) with B3LYP6-31G(d,p) mapped on the surface [in the range −0.062 to +0.130 atomic units; the red and blue regions represent negative and positive electrostatic potentials, respectively], (b) rescale the surface EP property to limit the range to ±0.025 au (c) the geometric norm function d_{norm} [from −0.5 (blue) to 0.5 Å (red)] mapped onto them, and (d) the shape index property [in the ranges −1 to 1 Å].

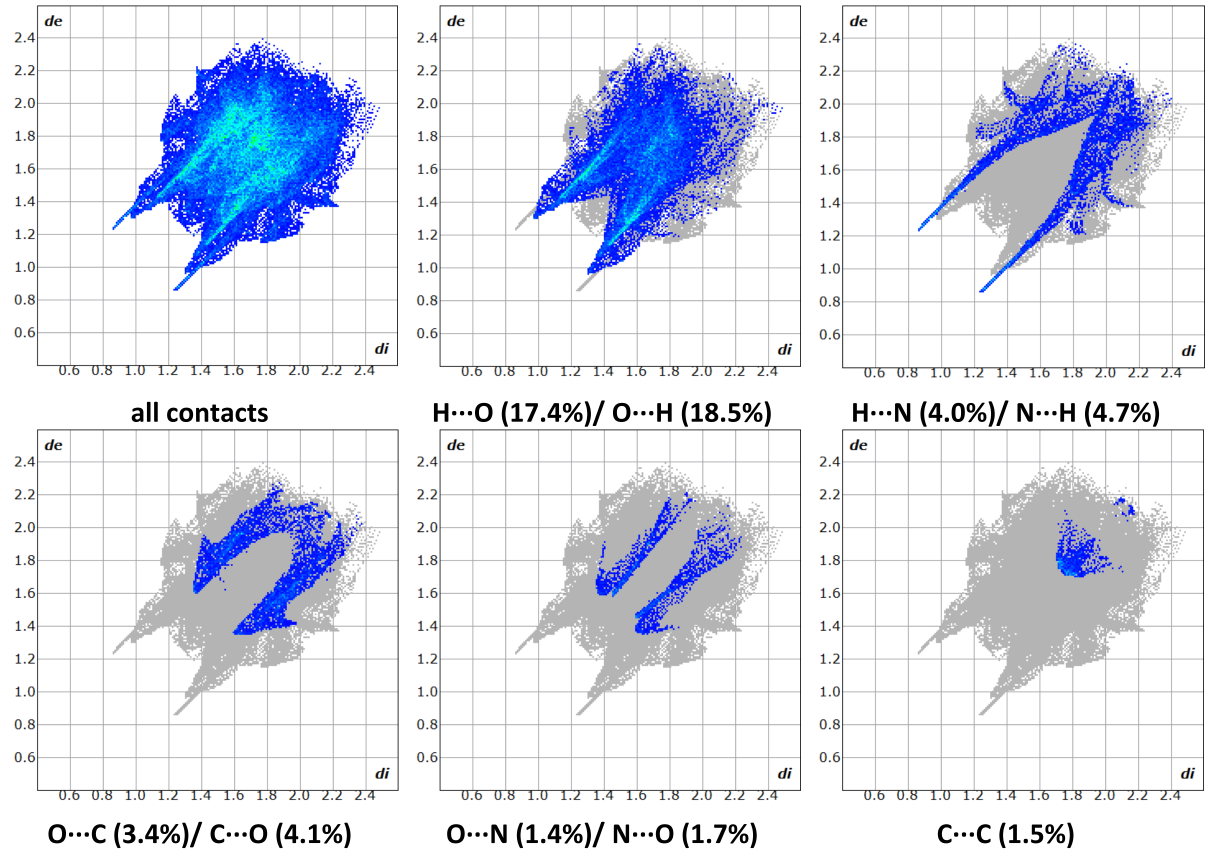

Fig. S2. 2D fingerprint plots with selected types of contacts and their contribution to the surface area for **4MDNPHP**.

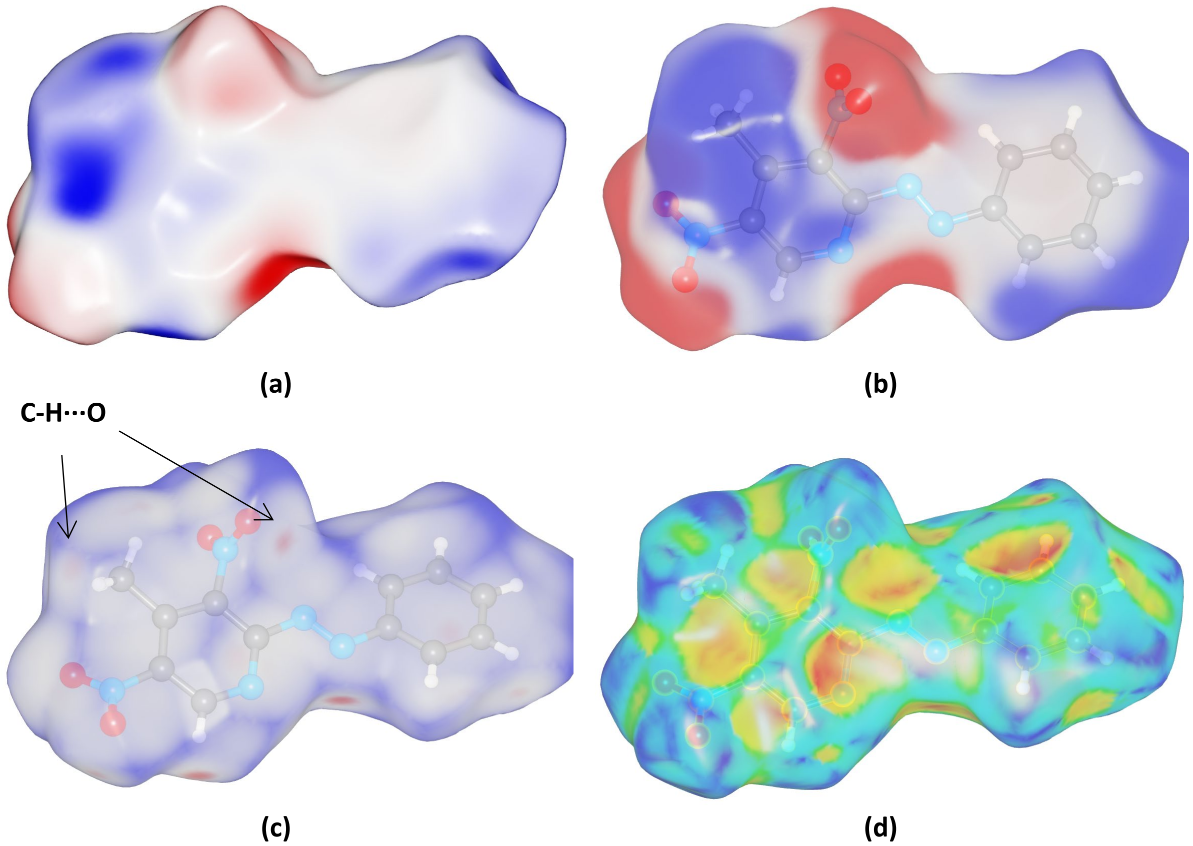

Fig. S3. Hirshfeld surfaces for **4MDNPAP** with: (a) the electrostatic potential (EP) with B3LYP6-31G(d,p) mapped on the surface [in the range −0.075 to +0.053 atomic units; the red and blue regions represent negative and positive electrostatic potentials, respectively], (b) rescale the surface EP property to limit the range to ±0.025 au (c) the geometric norm function d_{norm} [from −0.25 (blue) to 1 Å (red)] mapped onto them, and (d) the shape index property [in the ranges −1 to 1 Å].

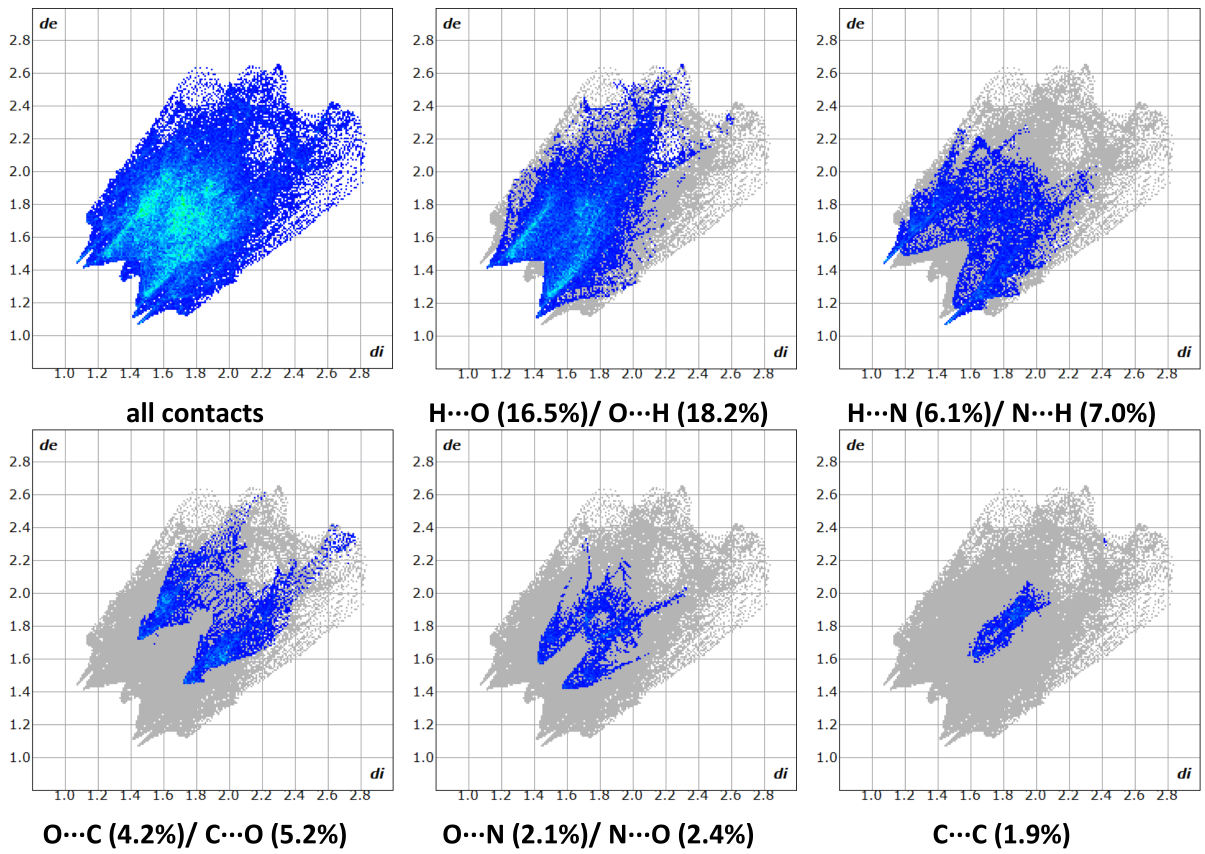

Fig. S4. 2D fingerprint plots with selected types of contacts and their contribution to the surface area for **4MDNPAP**.

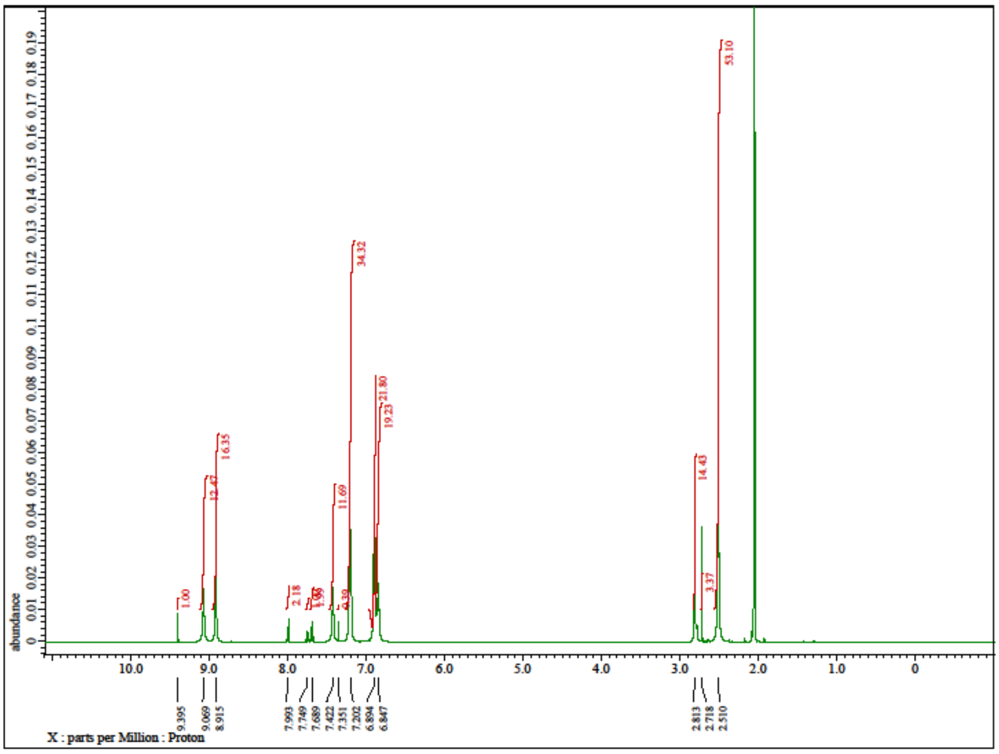

(a)

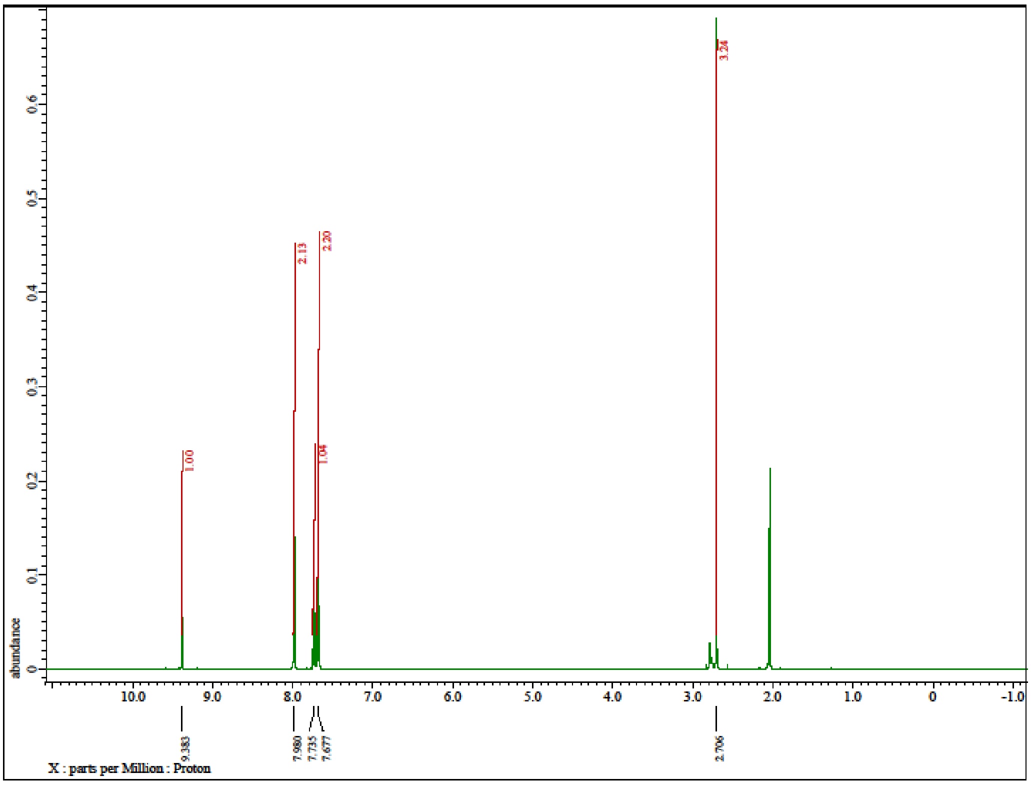

(b)

Fig. S5. ^{1}H NMR spectra of **4MDNPHP** (a) and **4MDNPAP** (b).

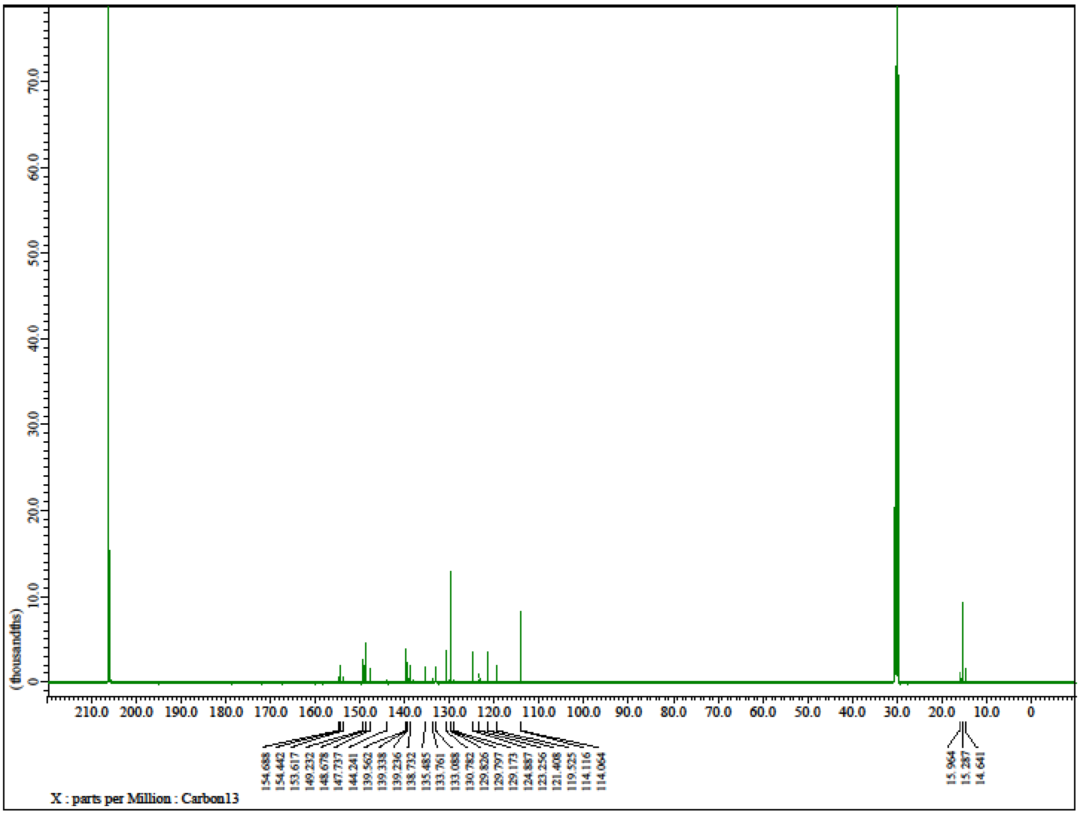

(a)

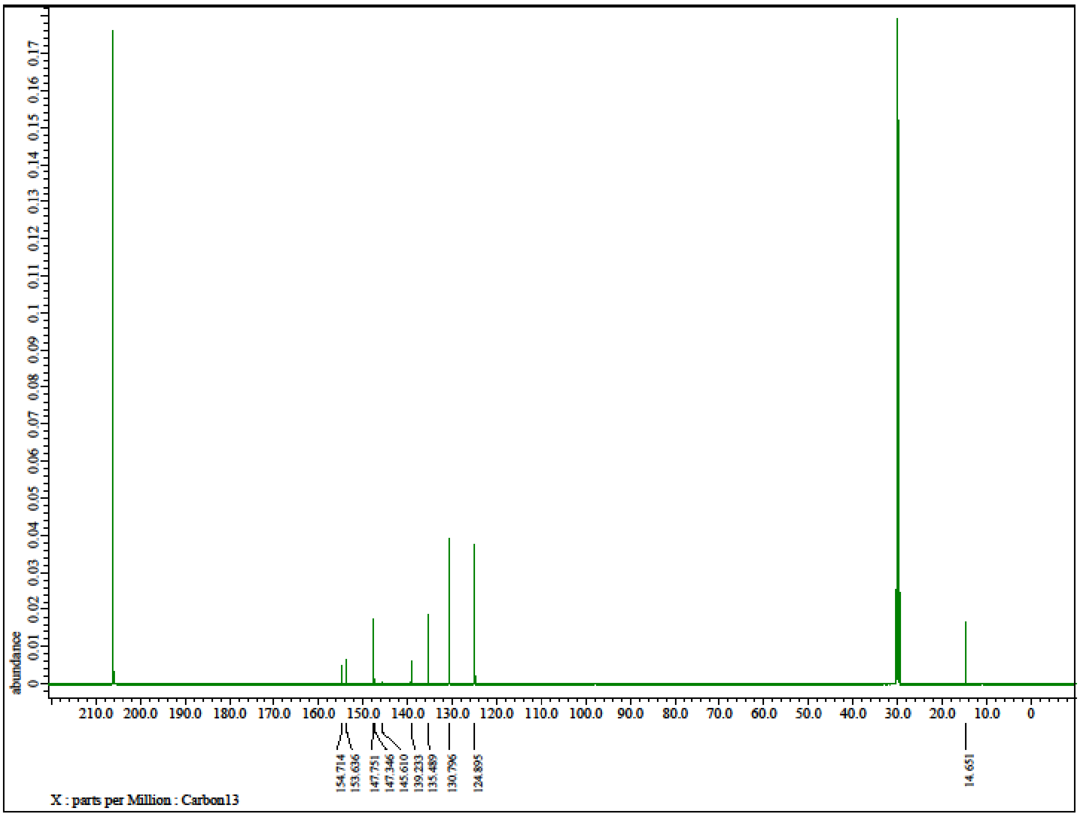

(b)

Fig. S6. ^{13}C NMR spectra of **4MDNPHP** (a) and **4MDNPAP** (b).

Table S2. Selected singlet and triplet electronic excited states, excitation energies, oscillator strange for **4MDNPHP**.

Electron levels Singlets (S) Triplets (T)	eV	nm	cm^{-1}	Oscillator strength f
T1	2.8015	442.57	22595	0.0000
T2	2.9205	424.53	23555	0.0000
T3	2.9870	415.08	24092	0.0000
T4	3.0329	408.80	24462	0.0000
S1	3.0653	404.48	24723	0.0371
T5	3.1570	392.72	25463	0.0000
S2	3.2370	383.02	26108	0.0526
T6	3.2935	376.45	26564	0.0000
T7	3.5321	351.02	28488	0.0000
S3	3.5748	346.83	28833	0.0139
T8	3.7069	334.47	29898	0.0000
T9	3.7560	330.10	30294	0.0000
T10	3.7823	327.80	30506	0.0000
S4	3.7944	326.75	30604	0.0442
S5	4.0982	302.54	33053	0.0079
S6	4.1802	296.60	33715	0.0236
S7	4.2039	294.92	33908	0.0318
S8	4.2500	291.73	34278	0.0124
S9	4.2952	288.66	34643	0.1058
S10	4.3245	286.70	34880	0.0009

Table S3. Selected singlet and triplet electronic excited states, excitation energies, oscillator strange for **4MDNPAP**.

Electron levels Singlets (S) Triplets (T)	eV	nm	cm^{-1}	Oscillator strength f
T1	1.5810	784.19	12752	0.0000
T2	2.2315	555.62	17998	0.0000
T3	2.8660	432.61	23116	0.0000
S1	2.2898	541.46	18469	0.0056
T4	3.0049	412.61	24236	0.0000
T5	3.0811	402.40	24851	0.0000
T6	3.1236	396.93	25193	0.0000
T7	3.2748	378.60	26413	0.0000
T8	3.3445	370.71	26975	0.0000
S2	3.3909	365.63	27350	0.5850
T9	3.4584	358.50	27894	0.0000
S3	3.5350	350.74	28511	0.0435
S4	3.5658	347.70	28760	0.0550
S5	3.6310	341.46	29286	0.0431
T10	3.6384	340.76	29346	0.0000
S6	3.7780	328.17	30472	0.0324
S7	3.8205	324.52	30815	0.0054
S8	3.8481	322.19	31038	0.0032
S9	4.0069	309.43	32318	0.0141
S10	4.2371	292.61	34175	0.0006

Table S4. Mulliken atomic charges calculated for **4MDNPHP**.

Atom number	Mulliken charges	Atom number	Mulliken charges
C1′	0.221218	N1	-0.427640
C2′	-0.090814	C6	0.197527
H2′	0.064325	H6	0.096653
C3′	-0.061391	C5	-0.023501
H3′	0.064489	C4	0.111293
C4′	-0.067430	C3	0.009640
H4′	0.060634	C7	-0.097696
C5′	-0.059283	H7(1)	0.072905
H5′	0.064275	H7(2)	0.084561
C6′	-0.087193	H7(3)	0.098562
H6′	0.050625	N5	0.429489
N2′	-0.293111	O4	-0.373708
H_N2'	0.175751	O3	-0.378973
N2	-0.193026	N3	0.398759
H2	0.142688	O1	-0.352551
C2	0.539842	O2	-0.376919

Table S5. Mulliken atomic charges calculated for **4MDNPAP**.

Atom number	Mulliken charges	Atom number	Mulliken charges
C1′	0.205801	C6	0.201980
C6′	-0.040851	H6	0.097479
H6′	0.076889	C5	0.001949
C5′	-0.073173	C4	0.103371
H5′	0.071616	C3	-0.008367
C4′	-0.029216	N5	0.427513
H7′	0.072339	O3	-0.368312
C3′	-0.072019	O4	-0.358652
H9′	0.068679	C7	-0.102463
C2′	-0.031116	H7(1)	0.074504
H2′	0.068533	H7(2)	0.087998
N2′	-0.198235	H7(3)	0.101394
N2	-0.297524	N3	0.412780
C2	0.588201	O1	-0.358502
N1	-0.364021	O2	-0.358575

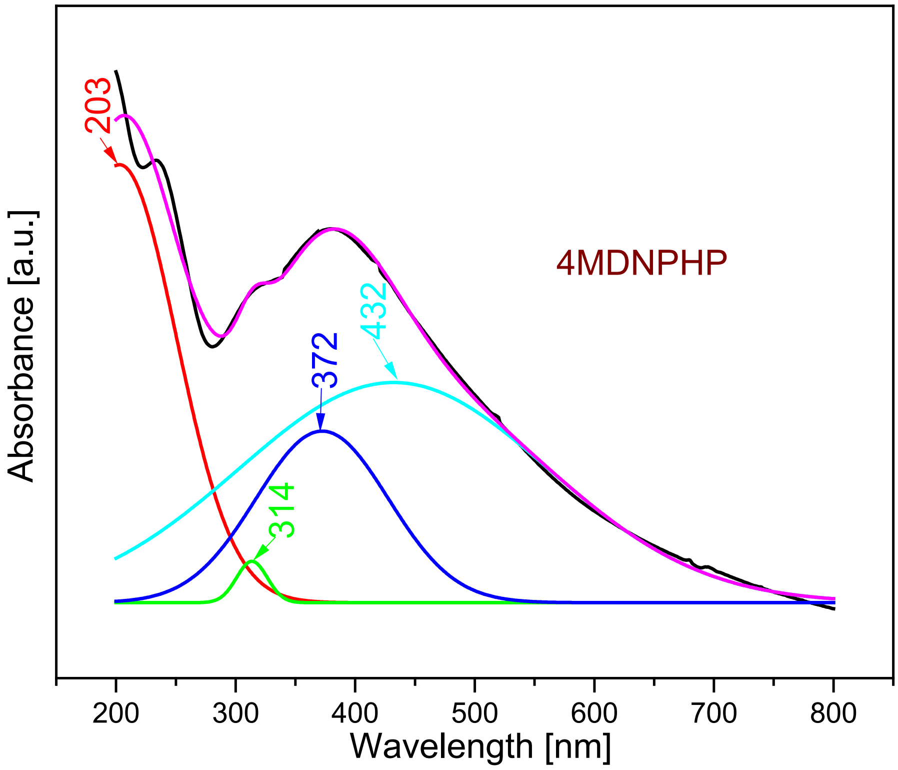

(a)

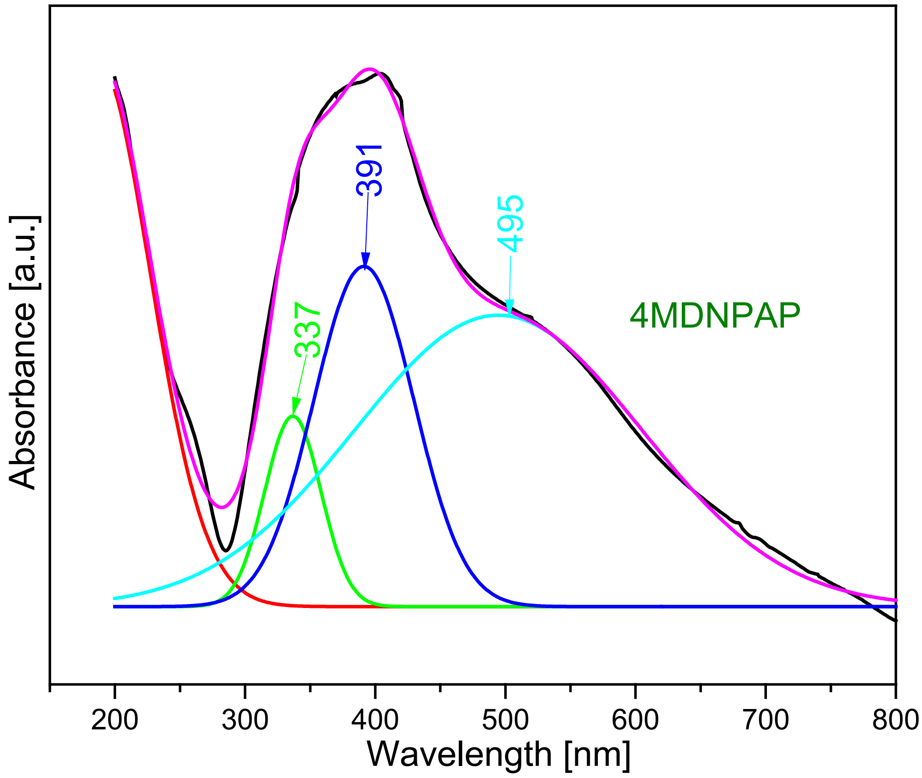

(b)

Figure S7. The Gauss deconvolution for the UV-Vis spectra of **4MDNPHP** (a) and **4MDNPAP** (b).
